# Systems engineering of *Escherichia coli* for high-level hydroxytyrosol production

**DOI:** 10.1016/j.synbio.2026.04.025

**Published:** 2026-06-01

**Authors:** Jiaojiao Zuo, Shaolun Zhang, Wenxiao Huang, Jia Liu, Cong Gao, Gui peng Hu, Wei Song, Xiaomin Li, Wanqing Wei, Jing Wu, Liming Liu, Kaifang Liu, Nan Xu

**Affiliations:** a College of Bioscience and Biotechnology, Yangzhou University, Yangzhou, China; b School of Biotechnology, Jiangnan University, Wuxi, 214122, China; c Key Laboratory of Industrial Biotechnology, Ministry of Education, Jiangnan University, Wuxi, 214122, China; d School of Life Sciences and Health Engineering, Jiangnan University, Wuxi, 214122, China

**Keywords:** Hydroxytyrosol, Metabolic flux imbalances, By-product elimination, Cofactor engineering, Hydroxytyrosol tolerance, Fermentation optimization

## Abstract

Hydroxytyrosol (HT) is a potent polyphenolic antioxidant widely utilized in the biomedical and food industries. However, its high-level microbial biosynthesis is primarily hindered by the metabolic flux imbalances and severe cellular toxicity. In this study, an artificial synthetic pathway from 4-hydroxyphenylpyruvate was constructed in an engineered l-phenylalanine producing *E*. *coli* chassis. Building on this, the endogenous precursor supply was strengthened via targeted promoter engineering of *aroK*, *aroC*, and *tyrA*, and the heterologous HT biosynthetic pathway was enhanced by overexpressing ARO10. To mitigate intermediate l-DOPA accumulation, co-expression of l-DOPA decarboxylase (DODC) and tyramine oxidase (TYO) reduced l-DOPA by 63.7%, while expression of l-amino acid deaminase (LAAD) reduced l-DOPA by 76.1%. Additionally, precise cofactor engineering was implemented; overexpressing the riboflavin metabolic genes *ribH*, *ribC*, and *ribF*, alongside introducing *pntAB*, increased HT production by 30.9% and 12.7%, respectively. Furthermore, transcriptomic analysis under HT stress revealed significant upregulation of genes related to transport and stress responses. Among these targets, overexpressing *marR* substantially improved cellular tolerance and HT production. Finally, during a 5-L bioreactor fermentation supplemented with Fe^2+^ and ascorbic acid, the engineered strain achieved an HT titer of 9.25 g/L, a yield of 0.102 g/g glucose, and a productivity of 0.193 g/L/h. This study reports the highest HT titer to date in *E. coli* using glucose as the carbon source, providing a robust biomanufacturing platform.

## 1 Introduction

Hydroxytyrosol (HT), also known as 3,4-dihydroxyphenylethanol, is a simple amphoteric phenol containing a catechol group and an ethanol side chain, making it a polar organic solvent that dissolves in both water and organic solvents. It is mostly obtained from olives and their processing byproducts [[1], [2], [3], [4]]. Because of its molecular structure, HT has strong antioxidant, anti-inflammatory, antibacterial, and anticancer properties, making it particularly desirable for usage in functional foods, pharmaceutical formulations, and high-end cosmetics [[5], [6], [7], [8]]. Due to the high market value of HT, which is expected to approach 2.5 billion USD by 2030 [9]. The primary techniques of producing HT are plant extraction, chemical synthesis, and microbial fermentation. Traditional extraction methods rely primarily on olive resources, which have a number of disadvantages, including strong regional dependence, limited extraction efficiency, considerable environmental impact, and variable raw material supply. In contrast, chemical synthesis frequently necessitates the use of hazardous reagents and harsh reaction temperatures, limiting its scalability and green development potential [[10], [11], [12]]. In contrast, biological synthesis for HT production has various benefits, including mild reaction conditions, process controllability, and great sustainability, making it an attractive strategy for the effective biomanufacturing of HT [[13], [14], [15]].

Biocatalysis has been widely employed in hydroxytyrosol synthesis, which validated many feasible synthetic enzymes and pathways for hydroxytyrosol production. The viability of microbial catalysis for HT synthesis was confirmed for the first time when a multi-enzyme pathway for the conversion of tyrosine to HT was built in *E. coli* using mammalian tyrosine hydroxylase and substituting the cofactor [16]. By replacing tyrosine hydroxylase with an engineered two-component flavin-dependent monooxygenase (HpaBC) and obtaining high-activity mutants (23F9-M4) through saturation mutagenesis, the titer of l-DOPA was increased by 15-fold. Additionally, a biosensor-based screening method was developed, leading to the identification of an efficient tyramine oxidase mutant (TYOYM9-2) that addressed the toxicity issue caused by the accumulation of the intermediate dopamine, ultimately increasing HT production to 4.69 g/L [17]. In another study, using l-DOPA as a substrate, a high conversion rate of 89.5% was achieved through a cascade of transamination, decarboxylation, and reduction reactions, resulting in 5.59 g/L of HT, with this pathway avoiding oxidative losses [18]. Recently, a highly efficient *E. coli* BL21 synthesis technique was devised employing 3,4-dihydroxybenzaldehyde and l-threonine as substrates, reaching the conversion of 30 mM 3,4-dihydroxybenzaldehyde to 28.7 mM HT [19]. Whole-cell catalysis, which involves the addition of substrates and cell collection, is not appropriate for industrial production. In contrast, HT biosynthesis from basic carbon sources has tremendous potential to lower manufacturing costs and is regarded as a more viable route for industrial-scale uses.

Currently, model microorganisms such as *Saccharomyces cerevisiae*, *E. coli*, and *Bacillus licheniformis* are employed in the *de novo* synthesis of HT. However, the HT synthesis performance of *S*. *cerevisiae* in a 5 L fermentation system is significantly limited, with a product yield of only 6.97 g/L, a production rate of 0.097 g/L/h, and a relatively long fermentation cycle [20]. In contrast, *E. coli* has been reported to produce 9.87 g/L of HT, with a production rate of 0.282 g/L/h [21]. *B*. *licheniformis* can produce 9.47 g/L of HT, with a production rate of 0.263 g/L/h [22]. In terms of overall product synthesis levels, production rates, and industrial application potential, *E. coli* is regarded as a suitable host for HT production due to its advantages, which include simplicity of genetic modification, a defined metabolic network, rapid growth, and extensive carbon source consumption. Metabolic engineering strategies for HT production aim to address four primary bottlenecks: insufficient precursor supply, low catalytic activity of key enzymes, feedback inhibition, and the toxicity of HT itself. To increase precursor supply, *ptsG* and *pykA* were knocked out, while *glf* and *glk* were overexpressed to boost the carbon source transport efficiency of the non-PTS system, hence increasing the supply of phosphoenolpyruvate (PEP) [21]; To enhance key enzyme activity, to endow HpaBC with hydroxylation activities toward both tyrosol and tyramine, site-directed mutagenesis was performed on HpaBC to generate mutant H7 (S210T/A211 M/Q212G). The catalytic activities of mutant H7 toward tyrosol and tyramine were increased by approximately 16-fold and 270-fold, respectively, enabling synergistic conversion via dual pathways. Using a fed-batch strategy, the production titer of hydroxytyrosol reached 1.89 g/L with a conversion rate of 82% [23]. A semi-rational design in conjunction with site-saturation mutagenesis was employed produce efficient mutants ARO10^D331V^ and ARO10^D331C^. Tyrosol, as an intermediate product had titers of 2.02 g/L and 2.04 g/L, respectively, which was more than 50% higher than that of the wild-type strain [24]. To alleviate feedback inhibition, the *aroG* encoding the 3-deoxy-d-arabino-heptulosonate-7-phosphate (DAHP) synthase and *tyrA* encoding the branched-chain amino acid aminotransferase are susceptible to feedback inhibition by tyrosine. Mutants of *aroG*^D146N^ and *tyrA*^M53I/A354V^ relieved this inhibition and further enhancing the precursor metabolic flux [22]. A method that combined the removal of ammonium chloride from the culture medium with the addition of dodecanol and ascorbic acid to reduce the toxicity of HT resulted in a 75% increase in the final HT yield when compared to the original media [25]. The HT biosynthesis capacity of engineered strains was greatly enhanced by the combined use of these metabolic engineering techniques; nevertheless, there is still much space for improvement in the iterative optimization of large-scale fermentation processes, which must be further improved to satisfy the requirements of industrial production.

**Fig. 1:**
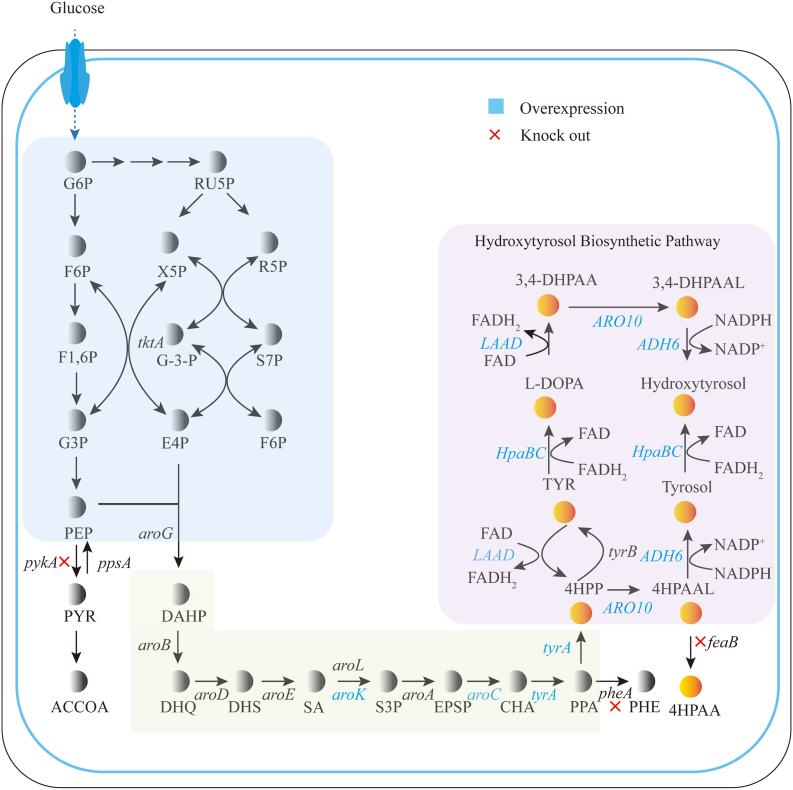
The function of each gene is as follows: *tktA*: Transketolase; *aroG*: 3-deoxy-d-arabino-heptulosonate-7-phosphate synthase; *ppsA*: Phosphoenolpyruvate synthase; *pykA*: Pyruvate kinase; *aroB*: 3-dehydroquinate synthase; *aroD*: 3-dehydroquinate dehydratase; *aroE*: Shikimate dehydrogenase; *aroK*: Shikimate kinase; *aroA*: 5-enolpyruvylshikimate-3-phosphate synthase; *aroC:* Branch chain acid synthase; *trpE*: Anthranilate synthase component I; *tyrA*: Prephenate dehydratase; *pheA*: Chorismate mutase; *ARO10*: Phenylpyruvate decarboxylase; *tyrB*: Aromatic amino acid transaminase; *LAAD*: l-amino acid deaminase; *ADH6*: Alcohol dehydrogenase; *HpaBC*: 4-hydroxyphenylacetic acid 3-monooxygenase; *feaB*: Phenylacetaldehyde dehydrogenase. Abbreviations: G6P: Glucose-6-phosphate; F6P: Fructose-6-phosphate; F1,6P: Fructose-1,6-bisphosphate; G3P: Glyceraldehyde-3-phosphate; PEP: Phosphoenolpyruvate; PYR: Pyruvate; ACCoA: Acetyl-CoA; RU5P: Ribulose-5-phosphate; X5P: Xylulose-5-phosphate; R5P: Ribose-5-phosphate; S7P: Sedoheptulose-7-phosphate; E4P: Erythrose-4-phosphate; 3 PG: 3-phosphoglycerate; DAHP: 3-deoxy-D-arabino-heptulosonate-7-phosphate; DHQ: 3-dehydroquinate; DHS: 3-dehydroshikimate; SA: Shikimate; S3P: Shikimate-3-phosphate; EPSP: 5-enolpyruvylshikimate-3-phosphate; CHA: Branch chain acids; PPA: Prephenate; 4HPP: 4-hydroxyphenylpyruvate; 4HPAAL: 4-hydroxyphenylacetaldehyde; 4HPAA: 4-hydroxyphenylacetic acid; l-DOPA: l-Dihydroxyphenylalanine; 3,4-DHPAA: 3,4-dihydroxyphenylpyruvate; 3,4-DHPAA L: 3,4-dihydroxyphenylacetaldehyde; TYR: Tyrosine; TRP: Tryptophan; PHE: Phenylalanine.

In this study, a hydroxytyrosol (HT) biosynthesis pathway was constructed in *E. coli* JNYPQ (Fig. 1), with strategies focused on enhancing precursor supply, strengthening the main synthesis pathway, eliminating by-products, constructing a cofactor cycle, improving strain tolerance, and optimizing fermentation. Key enzyme genes were integrated with high copy numbers, and promoter engineering was used to optimize the expression of the *aroK*, *aroC*, and *tyrA*^fbr^ genes, eliminating the accumulation of the precursor shikimate. The impact of blocking side pathways on HT synthesis was also analyzed. To reduce the accumulation of the intermediates l-DOPA, l-amino acid deaminase (LAAD) was introduced. Cofactor engineering was employed to further optimize HT biosynthesis by strengthening NADPH and FAD supply. Transcriptional analysis under HT stress revealed that overexpression of *marR* played a crucial role in enhancing strain tolerance and increasing HT production. Lastly, Fermentation process optimization using simple carbon sources in a 5 L fermenter achieved a HT titer of 9.25 g/L, with a glucose conversion rate of 0.102 g/g. This represents the highest reported HT yield to date in *E. coli* using glucose as the carbon source, providing new insights for microbial HT production.

## 2 Materials and methods

### 2.1 Strains and plasmids

*E. coli* JNYPQ was used as the host strain for hydroxytyrosol (HT) production. In this study, we used an l-phenylalanine-producing *E*. *coli* strain previously constructed in our laboratory (strain deposit number: GDMCC No. 62245, patent number: CN116656581A). This laboratory stock strain was obtained by mutagenesis and screening from the K12 strain. On this basis, the *tyrA* was complemented to generate the *E*. *coli* chassis strain JNYPQ used in this study. *E. coli* TO10 and DH5α were employed as cloning hosts for plasmid construction, while *E. coli* BL21(DE3) was used for protein expression. The strains and plasmids used in this study are listed in Tables S1 and S2, respectively. The promoters and promoter sequences used in this study are shown in Table S3. The primers and synthetic DNA sequences are provided in Table S4. Expression vectors pRSFDuet, pETDuet, pCDFDuet, and pACYCDuet were utilized for gene overexpression. Plasmid construction was performed using ClonExpress one-step cloning (Novozymes, Nanjing, China). Gene integration into the genome was achieved using the CRISPR/Cas9 editing system.

All heterologous genes employed in this study were commercially synthesized by Tianlin (Wuxi, China). These genes included: 4 hydroxyphenylacetate 3 hydroxylase HpaBC was derived from *E. coli* BL21 (GenBank ID: HpaB: ACT46003, HpaC: ACT46002), *Salmonella enterica* (GenBank ID: HpaB: CBW17134, HpaC: CBW17133), and *Klebsiella pneumoniae* (GenBank ID: HpaB: ABR80126, HpaC: ABR80125). Phenylpyruvate decarboxylases from different sources included SeaARO10 from *S. cerevisiae* (GenBank ID: CP029160.1), KpnPDC from *K*. *pneumoniae* (GenBank ID: ABR78156.1), and KapPDC from *Yarrowia lipolytica* (GenBank ID: XP_002493734). Alcohol dehydrogenases included ADH6 from *S*. *cerevisiae* (GenBank ID: 851987) and YahK from *E*. *coli* BL21 (GenBank ID: ACT42179.1). l-DOPA decarboxylase DODC was from *Pseudomonas putida* (GenBank ID: AAN68161), and tyramine oxidase TYO was from *Micrococcus luteus* (GenBank ID: LC604736.1). In addition, NADH kinase Pos5P from *S*. *cerevisiae* (GenBank ID: 855913) and glucose dehydrogenase GDH from *Bacillus subtilis* (GenBank ID: QEZ93655.1).

### 2.2 Media

In this study, *E. coli* strains were cultured in LB medium, which contains 10 g/L of tryptone, 5 g/L of yeast extract, and 10 g/L of sodium chloride. The seed medium consists of 5 g/L of sodium chloride, 10 g/L of yeast extract, 32 g/L of tryptone, and 10 g/L of glycerol. The fermentation medium contains 25 g/L of glucose, 7 g/L of yeast extract, 2 g/L of K_2_HPO_4_, 2.5 g/L of MgSO_4_·7H_2_O, 7.5 g/L of (NH_4_)_2_SO_4_, 10 g/L of glycerol, 2 g/L of sodium citrate, 1.2 g/L of phenylalanine, 1 g/L of thiamine hydrochloride, 0.1 mg/L of nicotinic acid, 1 mg/L of biotin, and 1 mL of metal ion solution per liter (containing 2.5 g/L of Na_2_MoO_4_·2H_2_O, 0.5 g/L of ZnSO_4_·7H_2_O, 0.125 g/L of H_3_BO_3_, 0.25 g/L of CuCl_2_·2H_2_O, 6 g/L of MnSO_4_·H_2_O, 30 g/L of FeSO_4_·7H_2_O, 1.75 g/L of CoCl_2_·6H_2_O, 2.5 g/L of NiSO_4_·6H_2_O, and 2.5 g/L of AlCl_3_·6H_2_O). The pH of both seed and fermentation media was adjusted to 6.60 before sterilization using ammonia solution. The glucose concentration for fed-batch culture was set at 800 g/L.

### 2.3 Cultivation conditions

Shake flask fermentation was conducted by culturing the *E. coli* strain on solid LB medium at 37 °C for 12 h. The cells were harvested by washing with 6 mL of LB medium, and 500 μL of the culture was inoculated into primary LB medium for further incubation at 37 °C with shaking at 220 rpm for 12 h. Subsequently, 1 mL of the primary seed culture was transferred to secondary seed medium and incubated at 37 °C with shaking at 120 rpm for 7 h. Finally, the secondary seed culture was inoculated into the fermentation medium at a 4% (v/v) inoculation ratio. When the OD_600_ reached 1.0, IPTG was added to a final concentration of 0.1 mM, and fermentation was carried out at 30 °C with shaking at 120 rpm for 48 h.

For the 5 L fermenter fermentation, the culture volume was maintained at 2.5 L with an air flow rate of 2.5 vvm and an inoculation ratio of 4%. The initial growth temperature was set to 37 °C, with the pH adjusted to 6.6 using 50% ammonia solution. The residual glucose level was maintained between 0 and 2 g/L by feeding glucose at a concentration of 800 g/L. When the OD_600_ reached 25, the fermentation temperature was lowered to 30 °C and stabilized for 30 min before adding 3 mL of a 100 mM IPTG solution. During the hydroxytyrosol fermentation process, to maintain a dissolved oxygen (DO) level of 30%, the initial agitation speed was set at 300 rpm, which was later increased to 800 rpm.

### 2.4 Whole-cell catalysis

The target gene fragments encoding 4-hydroxyphenylacetate 3-hydroxylase and phenylpyruvate decarboxylase from different sources were ligated with the pET28a expression vector harboring a T7 promoter, and the resulting homologous recombination products were transformed into *E*. *coli* BL21(DE3). *E. coli* BL21 cells harboring the target gene were cultured at 37 °C with 50 mg/L kanamycin. When the OD_600_ reached 0.6–0.8, IPTG was added to a final concentration of 0.1 mM to induce protein expression, and the cells were incubated at 25 °C for 16 h. Wet cells were collected by centrifugation and washed twice with PBS. The cells were disrupted by ultrasonication, and the supernatant after centrifugation was used as the crude enzyme extract. Subsequent reactions were performed using 20 g/L wet cells or crude enzyme extract. The reaction mixture (10 mL) containing 20 g/L wet cells or crude enzyme extract, PBS, and 10 mM tyrosol or 4-hydroxyphenylpyruvate was incubated at 37 °C for 1 h. The concentrations of HT (hydroxytyrosol) or tyrosol in the mixture were measured to evaluate the catalytic activities of 4-hydroxyphenylacetate 3-hydroxylase and Phenylpyruvate decarboxylase from different sources.

### 2.5 Transcriptomic analysis

Log-phase *E. coli* cells were treated with 3 g/L of hydroxytyrosol for 3 and 6 h, respectively. Afterward, the cells were centrifuged at 5000 rpm for 5 min at 4 °C, washed twice with PBS (pH 7.4), and then frozen in liquid nitrogen. The samples were sent to Genewiz for transcriptomic data analysis, with the untreated strain serving as the control.

### 2.6 Analytical methods

The residual glucose during fermentation was determined using the DNS colorimetric method. Biomass was monitored by measuring the OD at 600 nm using a spectrophotometer. Hydroxytyrosol (HT) and its by-products were quantified using high-performance liquid chromatography (HPLC; Thermo, USA). The analysis was performed using an Agilent ZORBAX SB-Aq C18 column (4.6 mm × 250 mm, 5 μm), with a flow rate of 1.0 mL/min and detection at 280 nm. The column temperature was set to 28 °C. A standard curve was established by preparing solutions of HT, tyrosol, tyrosine, and l-DOPA at concentrations of 0.1 g/L, 0.2 g/L, 0.3 g/L, 0.4 g/L, and 0.5 g/L. The mobile phase A consisted of 0.1% formic acid in water, and mobile phase B was 100% methanol. By-products such as shikimate, acetic acid, lactic acid, and pyruvic acid were analyzed using another HPLC system (Waters, USA) with an HPX-87H ion-exchange column (Bio-Rad). The mobile phase for organic acid analysis was 5 mM dilute sulfuric acid, and the injection volume was 10 μL. The detection wavelength was set to 210 nm with a UV detector, and the flow rate was 0.6 mL/min, with a column temperature of 60 °C.

### 2.7 Cell mortality assay

The samples were harvested and centrifuged at 8000 r/min for 2 min, followed by washing twice with PBS buffer. The cell suspension was then diluted with PBS buffer to an OD_600_ of approximately 0.2, and 5 μL of propidium iodide (PI) staining solution was added. The mixture was incubated in the dark for 20 min. Flow cytometry was performed at a rate of 600–1000 cells per second (excitation wavelength: 536 nm; emission wavelength: 617 nm) to record the cell counts and fluorescence intensities of 20,000 cells with intact or damaged cell membranes. Cell mortality was defined as the ratio of the number of cells with damaged cell membranes to the total number of detected cells.

## 3 Results

### 3.1 Construction of a *de novo* hydroxytyrosol-producing strain

**Fig. 2:**
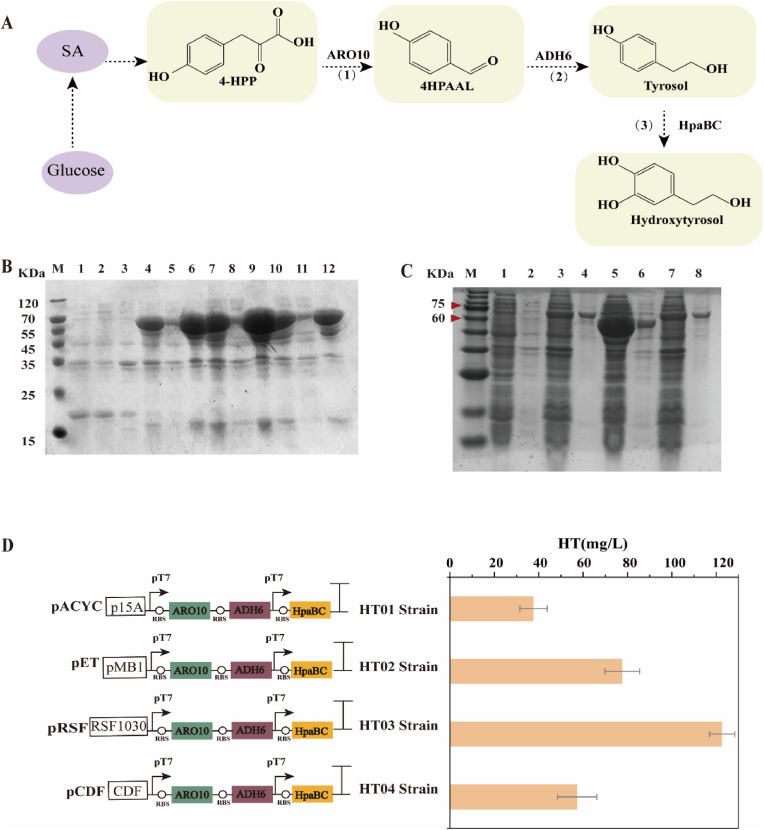
(A) Metabolic pathway diagram for hydroxytyrosol biosynthesis. (B) Expression levels of HpaBC proteins from different sources (SDS-PAGE analysis). 1: pET-28a empty vector control whole cells, 2: control supernatant, 3: control pellet, 4: EblHpaBC whole cells, 5: EblHpaBC supernatant, 6: EblHpaBC pellet, 7: KpnHpaBC whole cells, 8: KpnHpaBC supernatant, 9: KpnHpaBC pellet, 10: SenHpaBC whole cells, 11: SenHpaBC supernatant, 12: SenHpaBC pellet. (C) Expression levels of decarboxylases proteins from different sources (SDS-PAGE analysis). 1: control supernatant; 2: control pellet; 3: SeaARO10 supernatant; 4: SeaARO10 pellet; 5: KpnPDC supernatant; 6: KpnPDC pellet; 7: KapPDC supernatant; 8: KapPDC pellet. (D) Optimization of hydroxytyrosol production capacity through the synthesis pathway enzymes expressed under different plasmid copy numbers. Error bars represent the standard deviation from three independent experiments.

The previously engineered *E. coli* JNYPQ (GDMCC No. 62245) for phenylalanine production was used as the chassis to construct hydroxytyrosol biosynthetic modules. This strain could produce 4.12 g/l-phenylalanine during shake-flask cultivation, and after 48 h of fermentation in a 5-L bioreactor, the titer reached 35.6 g/L. The expression levels of genes encoding shikimate kinase (*aroK*, *aroL*), 5-enolpyruvylshikimate-3-phosphate synthase (*aroA*), and branch-chain acid synthase (*aroC*), were upregulated by 4.48, 5.95, 4.28, and 2.88-fold, respectively, compared to the starting strain (Fig. S1). These results indicate that the shikimate pathway in JNYPQ has a strong metabolic flux, essential for hydroxytyrosol production. Starting with 4-hydroxyphenylpyruvate (4-HPP), an intermediate metabolite of the shikimate pathway, the theoretically shortest route for producing hydroxytyrosol was introduced into strain JNYPQ. Hydroxytyrosolis produced through decarboxylation, reduction, and hydroxylation steps that arecatalyzed by phenylpyruvate decarboxylase, alcohol dehydrogenase, and 4-hydroxyphenylacetate 3-hydroxylase in order (Fig. 2A).

The target gene fragments encoding 4-hydroxyphenylacetate 3-hydroxylase and phenylpyruvate decarboxylase from different sources were ligated with the pET28a expression vector harboring a T7 promoter, and the resulting homologous recombination products were transformed into *E*. *coli* BL21. Hydroxylases (HpaBC) from *S*. *enterica* (Sen), *K*. *pneumoniae* (Kpn), and *E. coli* BL21 (Ebl) were assessed for catalytic activity (Fig. 2B). In crude enzyme assays, the three hydroxylases were able to convert 10 mM tyrosol into hydroxytyrosol at rates of 17.3%, 18.5%, and 20.1%, respectively. The three decarboxylases from *K*. *phaffii* (Kap), *K*. *pneumoniae* and *S. cerevisiae* (Sea) could be normally expressed in *E. coli* (Fig. 2C). The conversion of 10 mM 4-HPP to tyrosol was tested using whole-cell methods, with conversion rates of 20.9% (KapPDC), 21.9% (KpnPDC), and 25.3% (SeaARO10), respectively, demonstrating that *S. cerevisiae* decarboxylase had the highest activity. The alcohol dehydrogenases selected were ADH6 from *S*. *cerevisiae* and *YahK* from *E*. *coli* BL21. Three kinds of enzymes were expressed with dual-promoter plasmids (pACYCDuet-1), yielding recombinant plasmids pACYCDuet-*HpaBC*-*ARO10*-*ADH6* and pACYCDuet-*HpaBC*-*ARO10*-*Yahk*. These plasmids were introduced into *E. coli* BL21 cells to test their potential to convert 4-HPP to hydroxytyrosol. The conversion rates were 20.2% and 18.2%. The phenylpyruvate decarboxylase (ARO10) and alcohol dehydrogenase (ADH6) from *S. cerevisiae*, as well as the 4-hydroxyphenylacetate 3-hydroxylase (HpaBC) from *E. coli* BL21, were chosen for the final strain assembly based on their enzyme activity. Then, several copy plasmids such as pACYCDuet-1, pETDuet-1, pRSFDuet-1, and pCDFDuet-1 were used to express the biosynthetic module enzymes in JNYPQ, resulting in the formation of recombinant strains HT01-04. After 48 h of shake-flask cultivation, hydroxytyrosol production were 37 mg/L, 77 mg/L, 122 mg/L, and 57 mg/L for strains HT01-04, respectively. These results suggested that the high-copy plasmid pRSFDuet-1 was most favorable for hydroxytyrosol production (Fig. 2D).

To reduce carbon flux toward l-phenylalanine, *pheA* encoding chorismate mutase was deleted to generate strain HT05. After 48 h of fermentation, the hydroxytyrosol titer was 56 mg/L, and the biomass decreased dramatically. The decreased cell growth should be caused by inadequate aromatic amino acid production. Plasmid expression and IPTG stimulation might further raise the strain's metabolic burden. After adding 1.2 g/L phenylalanine to the medium, cell growth was restored and hydroxytyrosol rose to 150 mg/L after 48 h 4-HPP did not accumulate but was accompanied by 985 mg/L shikimate, indicating an inadequate endogenous precursor supply for hydroxytyrosol synthesis.

### 3.2 Engineering of the Hydroxytyrosol metabolic pathway

**Fig. 3:**
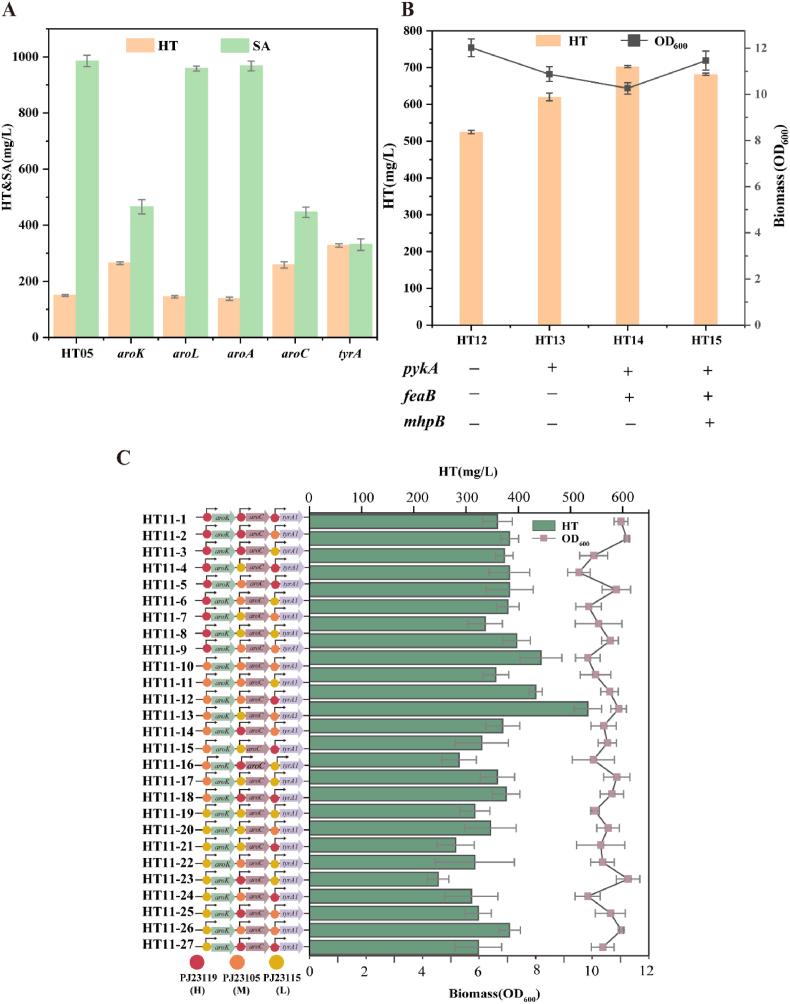
(A) Hydroxytyrosol production and shikimate accumulation in the shake-flask cultures of HT05 strains overexpressing *aroK*, *aroL*, *aroA*, *aroC*, and *tyrA1*, with HT05 as the control strain. (B) Growth and hydroxytyrosol production in shake-flask cultures of HT12 strains with sequential knockouts of *pykA* (HT13), *feaB* (HT14), and *mhpB* (HT15). (C) Expression balancing of *aroK*, *aroC*, and *tyrA1* through promoter engineering. Different promoter strengths were employed: H (high expression strength - PJ23119), M (medium expression strength - PJ23105), and L (low expression strength - PJ23115). Error bars represent the standard deviation from three independent experiments. Note: For the genes *pykA*, *feaB* and *mhpB*, the symbol "−" indicates the gene is not knocked out, and the symbol "+" indicates the gene is knocked out.

Shikimate kinase (*aroK*/*aroL*), 5-enolpyruvylshikimate-3-phosphate synthase (*aroA*), branch-chain acid synthase (*aroC*), and branch-chain acid aminotransferase (*tyrA*), are involved in the conversion of 4-hydroxyphenylpyruvate from shikimate. The strains HT06-10 were produced by overexpressing *tyrA*^M53I/A354V^ (*tyrA1*), *aroK*, *aroL*, *aroA*, and *aroC* in the genome of strain HT05. The overexpression of *aroL* and *aroA* boosted hydroxytyrosol production to 145 mg/L and 138 mg/L, respectively, with shikimate accumulation of 958 mg/L and 966 mg/L. Hydroxytyrosol production rose to 265 mg/L, 258 mg/L, and 327 mg/L with overexpression of *aroK* (HT06), *aroC* (HT09), and *tyrA1* (HT10), representing increases of 76.6%, 72.0%, and 118.0% over the HT05 strain. The shikimate accumulation of HT06 (*aroK*), HT09 (*aroC*) and HT10 (*tyrA1*) was reduced to 465, 446 and 331 mg/L, respectively, which indicated that the overexpression of these individual genes could effectively redirect the metabolic flux toward hydroxytyrosol biosynthesis (Fig. 3A).

Then three effective genes were assembled into the pJ01 plasmid, generating the recombinant plasmid pJ01-*aroK*-*aroC*-*tyrA1*. This plasmid was introduced into the HT05 strain to create HT11, which produced 460 mg/L hydroxytyrosol and 446 mg/L shikimate. To further reduce shikimate accumulation, expression levels of these genes were fine-tuned using three different promoters: PJ23119 (high expression, H), PJ23105 (medium expression, M), and PJ23115 (low expression, L). A total of 27 overexpression constructs were designed and introduced into *E. coli* HT05. The strain HT11-12(*aroK*(M)-*aroC*[M]-*tyrA1*[H]) achieved the highest hydroxytyrosol production (533 mg/L) among 27 combinations (Fig. 3C). This optimized construct was integrated into the genome of HT05 at the *ygaY* locus to generate strain HT12. HT12 produced 525 mg/L hydroxytyrosol, and shikimate accumulation was significantly reduced to 112 mg/L. These findings suggest that strengthening the endogenous precursor supply module (*aroK*-*aroC*-*tyrA1*) effectively reduces shikimate accumulation and promotes hydroxytyrosol production.

*PykA* encodes pyruvate kinase, which catalyzes the conversion of phosphoenolpyruvate (PEP) to pyruvate. The *pykA* deletion in HT12 strain increased the production of hydroxytyrosol to 620 mg/L, which might be related to PEP supply for shikimate pathway. The intermediate 4-hydroxyphenylacetaldehyde from the heterologous route can be oxidized to 4-hydroxyphenylacetic acid by *E. coli*'s endogenous phenylacetaldehyde dehydrogenase. After 48 h of shake-flask fermentation, the HT14 strain, generated by knocking out *feaB* in HT13, produced 703 mg/L hydroxytyrosol. Furthermore investigated was the deletion of the degradation gene *mhpB*, which has been proven to successfully lower hydroxytyrosol degradation [24]. Following *mhpB* deletion, the HT15 strain fermented in shake flasks for 48 h, yielding 682 mg/L hydroxytyrosol. Hydroxytyrosol production did not significantly increase as a result of *mhpB* deletion in this study (Fig. 3B).

### 3.3 Optimization of the Hydroxytyrosol synthesis module

**Fig. 4:**
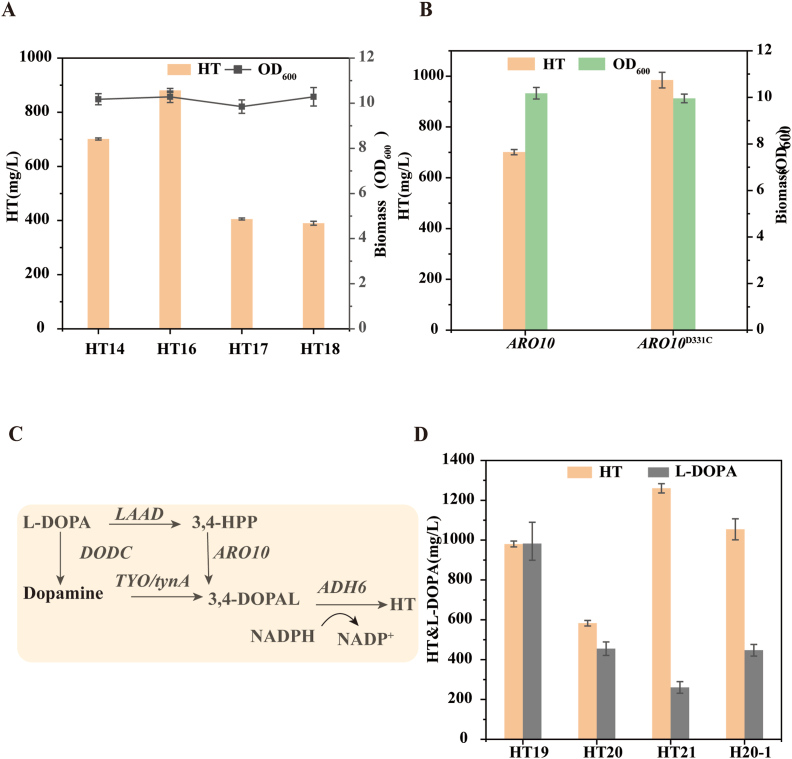
(A) Hydroxytyrosol production and growth in shake-flask cultures of HT14 strains overexpressing pathway enzymes gene *ARO10*, *ADH6*, and *HpaBC*, resulting in HT16, HT17, and HT18, respectively. (B) Hydroxytyrosol production and growth in shake-flask cultures of HT14 strains where the rate-limiting enzyme ARO10 was replaced with ARO10^D331C^. (C) Metabolic pathway diagram for the conversion of the byproduct l-DOPA to hydroxytyrosol. (D) Hydroxytyrosol production and l-DOPA accumulation in shake-flask cultures of HT19 strains with the introduction of DODC (HT20), LAAD (HT21), and both DODC and TYO (HT20-1). Error bars represent the standard deviation from three independent experiments.

To the heterologous hydroxytyrosol synthesis, the expression of enzymes in synthetic module was reinforced to drive more metabolic flux toward the intended product. Strains HT16–HT18 were produced by using the constitutive promoter PJ23119 to increase the expression of ARO10, ADH6, and HpaBC in the HT14 strain. According to shake-flask fermentation data, HT16, HT17, and HT18 produced 880 mg/L, 405 mg/L, and 390 mg/L hydroxytyrosol, respectively. The expression of phenylpyruvate decarboxylase (ARO10) enhanced hydroxytyrosol synthesis (Fig. 4A), which validated that phenylpyruvate decarboxylase was the key enzyme in the synthesis module. According to Xia et al., the ARO10^D331C^ mutant showed noticeably greater catalytic efficiency in hydroxytyrosol synthesis [26]. 984 mg/L hydroxytyrosol was produced by the HT19 strain, which was obtained by replacing *ARO10* with *ARO10*^D331C^ in the plasmid of the HT14 strain (Fig. 4B). Additional copies of *ARO10*^D331C^ were subsequently incorporated into the HT19 strain's genome, but no further increase in hydroxytyrosol production was seen.

The fermentation broth from HT19 after 48 h contains 987 mg/L l-DOPA because of the substrate promiscuity of the pathway enzymes ARO10 and HpaBC in the hydroxytyrosol synthesis module, which use l-tyrosine as a substrate in addition to converting 4-hydroxyphenylpyruvate. Following hydroxylation of l-tyrosine, l-DOPA spontaneously oxidizes to dopachrome and then polymerizes to create melanin in aerobic and mildly alkaline environments [27,28]. Genes that catalyze the conversion of l-DOPA were added to the hydroxytyrosol heterologous synthesis module in order to decrease the buildup of tyrosine and l-DOPA (Fig. 4C). The strains HT20 and HT21 were created by transforming the recombinant plasmids pRSFDuet-*HpaBC*-*DODC*-*ARO10*-*ADH6* and pRSFDuet-*HpaBC*-*LAAD*-*ARO10*-*ADH6* into the HT19 strain, which lacked the plasmid. The HT20 strain produced 583 mg/L hydroxytyrosol and accumulated 390 mg/L l-DOPA after 48 h of shake-flask fermentation. Whereas the HT21 strain produced 1.26 g/L hydroxytyrosol and accumulated 236 mg/L l-DOPA. l-DOPA was decarboxylated to dopamine by *P*. *putida* l-DOPA decarboxylase (DODC), which was expressed in HT19. The low capacity of *E. coli* native amine oxidase (*tynA*) to further oxidize dopamine to 3,4-dihydroxyphenylacetaldehyde (DOPAL) may be the cause of the decrease in hydroxytyrosol synthesis. *M*. *luteus* Tyramine oxidase (TYO) was expressed in the HT20 strain using the pET vector to increase the production of hydroxytyrosol, resulting in strain HT20-1. This strain accumulated 358 mg/L of l-DOPA and generated 1.05 g/L hydroxytyrosol (Fig. 4D). Furthermore, HT21 expressed *P*. *myxofaciens* l-amino acid deaminase (PmLAAD), which catalyzed the oxidation of l-DOPA at the amino group's 2-position to create 3,4-dihydroxyphenylpyruvate. ARO10 then decarboxylated this compound to create 3,4-dihydroxyphenylacetaldehyde (DOPAL), which was cut down to hydroxytyrosol. The PmaLAAD exhibits broad substrate specificity, oxidizing phenylalanine, tyrosine, and other amino acids. A portion of the accumulated tyrosine was oxidized to 4-hydroxyphenylpyruvate by PmaLAAD, which was then converted into hydroxytyrosol [29]. When l-amino acid deaminase (LAAD) was directly expressed, More hydroxytyrosol was produced under the direct expression of l-amino acid deaminase than the co-expression of l-DOPA decarboxylase (DODC) and Tyramine Oxidase (TYO).

### 3.4 Cofactor engineering optimizes hydroxytyrosol synthesis

**Fig. 5:**
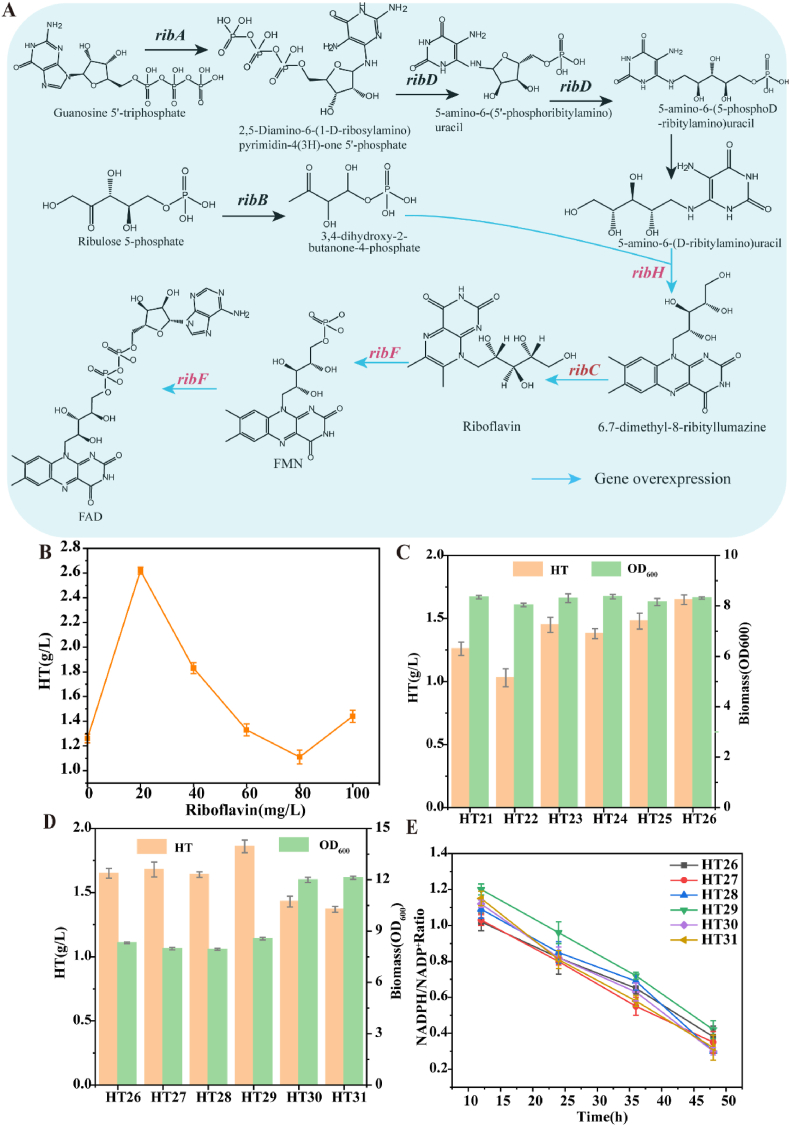
(A) Schematic of FAD synthesis in *E*. *coli*. (B) Effect of exogenous riboflavin supplementation at different concentrations on hydroxytyrosol production in shake-flask cultures of the HT21 strain. (C) Comparison of hydroxytyrosol production and growth after 48 h of shake-flask fermentation in HT21 and strains HT21–HT26, which overexpressed *guaA*, *ribH*, *ribC*, *ribF*, and *ribHCF*. (D) Effect of overexpression of *Pos5P*, *nadk*, *pntAB*, *GDH*, and *zwf* on hydroxytyrosol production and strain growth in HT26. (E) Changes in the NADPH/NADP^+^ ratio during 48 h of shake-flask fermentation for strains HT26–HT31. Error bars represent the standard deviation from three independent experiments.

NADPH/NADP^+^ and FADH_2_/FAD directly function as cofactors for many synthetic enzymes in the HT21 strain's hydroxytyrosol production module. In particular, l-amino acid deaminase PmaLAAD employs FAD as a cofactor, and 4-hydroxyphenylacetate 3-monooxygenase HpaBC uses FADH_2_ for the hydroxylation step. The intracellular redox balance may be upset by the high-throughput operation of the heterologous synthesis module, and the efficient expression of the heterologous synthesis module is negatively impacted by inadequate endogenous cofactor generation [30]. Exogenous riboflavin (0–100 mg/L) was added to shake-flask fermentation (Fig. 5B). The production of hydroxytyrosol increased when riboflavin was gradually added, peaking at 2.62 g/L when 20 mg/L riboflavin was added. This represents a 107.9% increase over the control strain. This finding implies that hydroxytyrosol production was much enhanced by the appropriate supply of FAD(H_2_) precursors via riboflavin.

*GuaA*, which catalyzes GMP synthesis, the precursor step in riboflavin production, was overexpressed in the HT21 strain to create the HT22 strain in order to increase the availability of FAD(H_2_) through riboflavin metabolism. Nevertheless, no significant improvement in hydroxytyrosol production was observed in HT22, which indicated that the downstream conversion of riboflavin to FAD might be the rate-limiting step in the riboflavin metabolic pathway. *RibH*, *ribC*, and *ribF*, which are involved in the four-step reaction from 5-Amino-6-(d-ribitylamino) uracil and 2-Hydroxy-3-oxobutyl phosphate to FAD (Fig. 5A), were expressed in strains HT23–25 based on the *E. coli* FAD production pathway. In comparison to the control strain, overexpression of *ribH*, *ribC*, and *ribF* resulted in hydroxytyrosol yields of 1.45 g/L, 1.38 g/L, and 1.48 g/L, respectively. These yield increases were 15.1%, 9.5%, and 17.4%. When the three targets were combined, the HT26 strain was obtained, with hydroxytyrosol production increasing to 1.65 g/L, a 30.9% increase over the control strain HT21 (Fig. 5C).

Alcohol dehydrogenase ADH6 requires NADPH to provide reducing power. To enhance the intracellular availability of NADP(H), two strategies were employed: (1) the introduction of a cofactor conversion system, and (2) expansion of the intracellular cofactor pool. In strategy (1), three key enzymes were selected for analysis: catalyzes the phosphorylation of NAD^+^ to NADP^+^; NADH kinase (Pos5P) from *S*. *cerevisiae*, which NAD^+^ kinase (encoded by *nadK*), which promotes the conversion of NADH to NADPH; and pyridine nucleotide transaminase (encoded by *pntAB*), which catalyzes the conversion of NADH to NADPH [31]. The results showed that, compared with the starting strain HT26, strain HT29 overexpressing the native *pntAB* exhibited a significantly increased hydroxytyrosol titer of 1.86 g/L, representing a 12.7% improvement. In contrast, overexpression of *Pos5P* from *S. cerevisiae* (strain HT27) and the native *nadK* (strain HT28) resulted in hydroxytyrosol titers of 1.68 g/L and 1.64 g/L, respectively, with no significant enhancement in product biosynthesis. In strategy (2), attempts were made to introduce heterologous enzymes or modify the native *E. coli* glycolysis pathway to enhance NADPH regeneration. Since *E. coli* lacks a typical glucose dehydrogenase (GDH), *B*. *subtilis* GDH was introduced, resulting in the HT30 strain. Additionally, *E. coli* native glucose-6-phosphate dehydrogenase (*zwf*) was overexpressed in the HT31 strain. Although overexpression of *zwf* or introduction of heterologous *GDH* improved the growth of the strains, hydroxytyrosol production actually decreased, with yields of 1.43 g/L and 1.37 g/L, respectively, representing a decrease of 13.3% and 16.9% compared to the control strain (Fig. 5D). Further analysis of the cofactor ratios during shake-flask fermentation supported these results. After 48 h of fermentation, the NADPH/NADP^+^ ratio in HT29 was increased by 10.2% compared to the control strain, while the NADPH/NADP^+^ ratios in HT27, HT28, HT30, and HT32 were similar to that of the control strain (Fig. 5E).

### 3.5 Screening and evaluation of Hydroxytyrosol tolerance targets

**Fig. 6:**
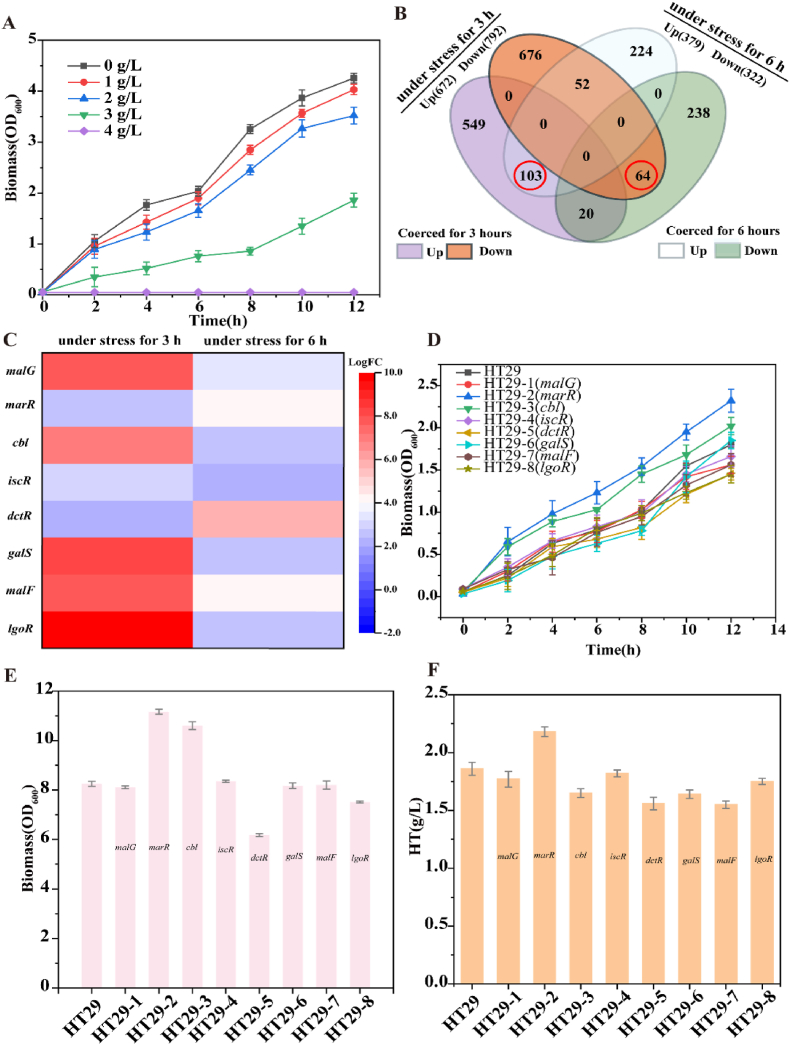
(A) Growth curve of HT29 strain in shake-flask cultures at different hydroxytyrosol concentrations. (B) Venn diagram of gene expression differences. (C) Abundance of selected candidate genes at the transcript level. (D) Growth of HT29-1 and HT29-8 under 3 g/L hydroxytyrosol stress. (E) Growth of strains HT29-HT29-8 in shake-flask cultures. (F) Hydroxytyrosol production by strains HT29-HT29-8 in shake-flask cultures. Error bars represent the standard deviation from three independent experiments.

When compared to the chassis strain JNYPQ, the modified hydroxytyrosol-producing strain HT29 showed a notable decrease in biomass. The biomass, as determined by OD_600_, was significantly reduced throughout fermentation, peaking at 28 h and then progressively decreasing after that. By the conclusion of fermentation, the mortality rate had increased to 32.2%, demonstrating a strong link with fermentation length (Fig. S2). The decrease in biomass and the rise in mortality indicate that hydroxytyrosol toxicity may prevent bacterial development and even cause cell death. The effects of exogenously added hydroxytyrosol at different concentrations (0 g/L, 1 g/L, 2 g/L, 3 g/L, 4 g/L) on the growth of HT29 strain were investigated. According to the results, the maximum biomass of HT29 was only 1.86 when 3 g/L hydroxytyrosol was applied, which was 56.23% less than that of the control strain (Fig. 6A). These results suggest that increasing the strain's tolerance to hydroxytyrosol could be a tactic to increase its capacity to produce hydroxytyrosol.

High-throughput RNA sequencing (RNA-seq) was used to evaluate gene expression in the HT29 strain under normal conditions and 3 g/L hydroxytyrosol stress in order to find genetic targets for improving hydroxytyrosol resistance. Compared with the untreated control, 1464 genes in the HT29 strain showed significant transcriptional changes under 3 g/L hydroxytyrosol stress for 3 h and 1001 genes exhibited significant alterations in transcription levels after 6 h of stress. 672 genes were considerably elevated and 792 genes were significantly downregulated following 3 h of stress; 379 genes were upregulated and 322 genes were downregulated following 6 h. A total of 103 genes were considerably upregulated and 64 genes were significantly downregulated as the duration of stress increased (Fig. 6B). Transporters, ferric enterobactin production, flagellar biosynthesis, and stress response were the main pathways associated with the genes showing notable alterations under both 3-h and 6-h hydroxytyrosol treatments. A total of eight genes associated with substrate transport were identified to be significantly upregulated at the transcriptome level. *MalG*, *marR*, *cbl*, *iscR*, *dctR*, *gals*, *malF*, and *lgoR* were chosen for validation based on this transcriptome analysis, with an emphasis on those with a fold change larger than 2 (Fig. 6C).The functions of these eight genes are as follows: *malG,* a membrane subunit of the maltose ABC transporter; *marR*, a DNA-binding transcriptional repressor; *cbl*, a DNA-binding transcriptional activator; *iscR*, a dual DNA-binding transcriptional regulator; *dctR*, a DNA-binding transcriptional regulator; *galS*, a dual DNA-binding transcriptional regulator; *malF*, a membrane subunit of the ABC transporter; and *lgoR*, a DNA-binding transcriptional regulator. HT29-1 through HT29-8 were produced by overexpressing these eight genes in HT29 using the potent promoter PJ23119. The experimental results of systematically evaluating the hydroxytyrosol tolerance of strains HT29-1 to HT29-8 under 3 g/L hydroxytyrosol stress showed that, compared with the control strain HT29, the growth of HT29-2 (*marR*) and HT29-3 (*cbl*) increased by 28.9% and 13.8% (Fig. 6D). Overexpression of *marR* and *cbl* significantly improved the hydroxytyrosol tolerance of the strain. These strains' capacity to grow and produce was rigorously assessed. According to shake flask fermentation results, overexpressing *marR* and *cbl* improved growth by 35.3% and 28.6%, respectively, in comparison to the control strain HT29, while other strains did not exhibit any discernible changes in growth (Fig. 6E). The *marR* overexpressing strain HT29-2's hydroxytyrosol production rose to 2.18 g/L after 48 h of shake-flask fermentation, which was 17.2% more than the control strain. In contrast, the other modified strains' hydroxytyrosol production did not significantly alter (Fig. 6F). These findings suggest that *marR* is essential for increasing the strain's ability to produce hydroxytyrosol by improving its tolerance to high hydroxytyrosol concentrations. In conclusion, overexpression of *marR* greatly increased hydroxytyrosol production, decreased cell mortality, and increased strain HT29-2's hydroxytyrosol tolerance. These results offer important information for creating industrial strains that produce hydroxytyrosol.

### 3.6 Fermentation optimization

**Fig. 7:**
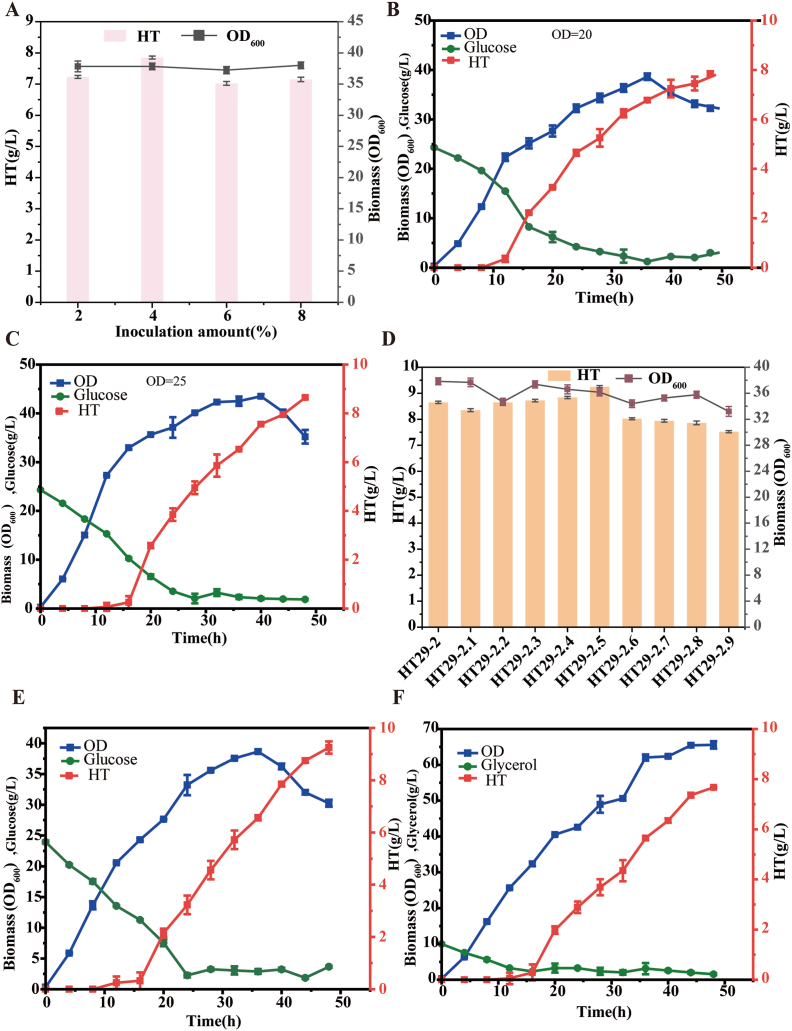
Fermentation optimization for hydroxytyrosol production in a 5 L fermenter. (A) Effect of different inoculum sizes on hydroxytyrosol production and cell growth. (B, C) IPTG induction of *E. coli* HT29-2 in a 5 L fermenter at OD_600_ = 20 and OD_600_ = 25, respectively, and analysis of fermentation parameters under different OD inductions. (D) Impact of varying Fe^2+^ and ascorbic acid concentrations on hydroxytyrosol production and cell growth in the HT29-2 strain. (E) De novo biosynthesis of hydroxytyrosol by the HT29-2 strain under optimal conditions with glucose as the carbon source. (F) De novo biosynthesis of hydroxytyrosol by the HT29-2 strain under optimal conditions with glycerol as the carbon source. Error bars represent the standard deviation from three independent experiments.

Four distinct inoculation ratios (2%, 4%, 6%, and 8%) were tested in a 5 L fermenter to investigate the impact of inoculum size on hydroxytyrosol synthesis, while maintaining consistent fermentation conditions. The best inoculum size, according to the data, was 4%, which produced 7.85 g/L hydroxytyrosol at a conversion rate of 0.082 g/g and volumetric productivity of 0.163 g/L/h. Hydroxytyrosol titers of 7.23 g/L, 7.02 g/L, and 7.15 g/L were produced by inoculum sizes of 2%, 6%, and 8%, respectively; conversion rates were 0.077 g/g, 0.078 g/g, and 0.074 g/g, and productivities were 0.151 g/L/h, 0.146 g/L/h, and 0.148 g/L/h (Fig. 7A). An extended lag phase delayed the onset of product formation at low inoculum sizes, whereas inoculum sizes greater than 4% resulted in excessive cell density, increased nutrient competition, decreased substrate utilization efficiency, and, ultimately, decreased hydroxytyrosol yield and conversion efficiency.

Fed-batch fermentations were conducted with different IPTG induction times but with comparable medium composition and temperature. The hydroxytyrosol production patterns of strain HT29-2 in a 5 L fermenter following the addition of 0.1 mM IPTG at OD_600_ values of 20 and 25 are shown in Fig. 7B and C. The hydroxytyrosol titer rose to 7.85 g/L with an average productivity of 0.163 g/L/h and conversion rate of 0.082 g/g when IPTG was added at OD_600_ = 20. By contrast, induction at OD_600_ = 25 produced a 0.086 g/g conversion rate, a 10.4% increase in productivity to 0.180 g/L/h, and a 10.1% increase in hydroxytyrosol titer to 8.65 g/L. These results demonstrate that IPTG induction at OD_600_ = 25 is more beneficial for the formation of hydroxytyrosol.

Transcriptomic analysis revealed that genes associated with Fe–S cluster assembly were significantly upregulated under hydroxytyrosol production stress, suggesting that Fe^2+^ plays an important role in hydroxytyrosol biosynthesis. The active site of the hydroxylase HpaBC requires Fe^2+^ as a catalytic cofactor, and maintaining an appropriate Fe^2+^ concentration during fermentation is a key factor for achieving high-level hydroxylation activity [32]. Furthermore, the inclusion of antioxidants is essential for reducing product loss since hydroxytyrosol is susceptible to oxidative destruction under aerobic conditions, while the hydroxylation reaction catalyzed by HpaBC requires molecular oxygen [31]. Thus, this study evaluated the effects of fed-batch supplementation with different concentration ratios of Fe^2+^ and ascorbic acid following IPTG induction on hydroxytyrosol production and cell growth (Table 1). The hydroxytyrosol titer rose to 9.25 g/L (a 6.5% improvement) and the conversion rate increased to 0.102 g/g (a 15.6% improvement) when 40 mg/L Fe^2+^ and 2 g/L ascorbic acid were added. However, the hydroxytyrosol titer declined to 7.63 g/L and the conversion rate reduced to 0.078 g/g when the Fe^2+^ concentration was raised to 50 mg/L while keeping ascorbic acid at 2 g/L. This is probably because excess Fe^2+^ has deleterious effects on cell growth. The hydroxytyrosol titer decreased to 7.25 g/L with a conversion rate of 0.081 g/g when the Fe^2+^ concentration was fixed at 40 mg/L and the ascorbic acid concentration was raised to 3 g/L, suggesting that too much ascorbic acid was also harmful to hydroxytyrosol production (Fig. 7D).

**Table 1:** The ratio of Fe^2+^ to ascorbic acid in different fermentation batches.

Fermentation batch	Fe^2+^ addition amount	ascorbic acid
HT29-2.1	30 mg/L	1 g/L
HT29-2.2		2 g/L
HT29-2.3		3 g/L
HT29-2.4	40 mg/L	1 g/L
HT29-2.5		2 g/L
HT29-2.6		3 g/L
HT29-2.7	50 mg/L	1 g/L
HT29-2.8		2 g/L
HT29-2.9		3 g/L

Previous studies have reported that glycerol supplementation can lead to higher hydroxytyrosol production compared with glucose [21]. This study examined how hydroxytyrosol production was affected by the use of either glucose or glycerol as the feeding carbon source. After 48 h of fermentation, strain HT29-2 produced 9.25 g/L of hydroxytyrosol with a conversion rate of 0.102 g/g when glucose was utilized as the carbon source (Fig. 7E). The feeding mixture was then supplemented with glycerol instead of glucose. In these circumstances, strain HT29-4's hydroxytyrosol titer dropped to 7.76 g/L after 48 h, with a conversion rate of 0.081 g/g (Fig. 7F). The biomass of strain HT29-2 increased from 42.5 to 62.3 when glycerol was utilized as the carbon source as opposed to glucose-fed conditions; however, the hydroxytyrosol titer and carbon source conversion rate dropped by 16.1% and 20.6%, respectively.

## 4 Discussion

In this study, a systematic metabolic engineering strategy was employed to achieve efficient biosynthesis of hydroxytyrosol (HT) in *Escherichia coli*. First, multigene combinatorial regulation coupled with promoter engineering was applied to optimize precursor supply, systematically strengthening the shikimate pathway and blocking competitive branches, thereby significantly improving precursor availability. To address the growth-inhibitory effect of hydroxytyrosol on the host strain, transcriptome sequencing was performed to identify *marR*, a key regulatory gene associated with HT-responsive tolerance. Finally, through synergistic optimization of cofactor regeneration and fermentation stability, the optimal engineered strain produced 9.25 g/L HT in a 5 L bioreactor within 48 h, with a glucose conversion rate of 10.2%, representing a remarkable improvement in titer for this synthetic pathway.

The precursor synthesis pathway's buildup of non-precursor chemicals suggests that 4-hydroxyphenylpyruvate synthesis capabilities can be further improved. First, phenylalanine and chorismite, the direct precursor of hydroxytyrosol, were not in competition when *pheA* was knocked out [33]. This study significantly increased the supply of 4-hydroxyphenylpyruvate by enhancing the expression of key nodes in the shikimate pathway (*aroK*, *aroC*, *tyrA*1), which elevated the HT yield from an initial 150 mg/L to 525 mg/L, while reducing the accumulation of shikimate by more than 80%. Compared with previous studies, we not only improved pathway flux through single-gene overexpression, but also achieved fine-tuned regulation of multi-gene expression via promoter engineering, thereby further optimizing the efficiency of the precursor supply module.This strategy is consistent with that reported by Wang et al. for enhancing tyrosine supply; nevertheless, our study systematically addressed the issues of precursor competition and flux partitioning through combinatorial regulation of multiple key genes [34].

In the hydroxytyrosol biosynthetic pathway, ARO10 and HpaBC exhibit substrate promiscuity and can convert l-tyrosine into l-DOPA. Under aerobic and neutral-to-alkaline conditions, l-DOPA readily undergoes oxidative polymerization to form melanin, which results in carbon flux loss and interferes with the synthesis of the target product. To address this issue, two engineering strategies were adopted in this study:(1) Co-expression of *P*. *putida* DODC and *M*. *luteus* TYO, leading to a hydroxytyrosol titer of 1.05 g/L; (2) Introduction of *Proteus mirabilis* LAAD, which oxidizes l-DOPA into 3,4-dihydroxyphenylpyruvate and redirects it back into the synthetic pathway, while expanding the substrate utilization range.Consequently, the titer of the final strain HT21 increased to 1.26 g/L, with the residual l-DOPA decreased to 236 mg/L. Future work may focus on protein engineering of LAAD to further reduce the accumulation of intermediate metabolites.

The biosynthesis of hydroxytyrosol (HT) consumes NADPH and FADH_2_, and insufficient cofactor supply represents one of the major bottlenecks limiting efficient HT production.In this study, overexpression of the riboflavin biosynthetic genes *ribH*, *ribC*, and *ribF*, together with introduction of the pyridine nucleotide transhydrogenase genes *pntAB*, significantly enhanced intracellular availability of FADH_2_ and NADPH, further increasing the HT titer to 1.86 g/L.Notably, overexpression of glucose dehydrogenase (GDH) or glucose-6-phosphate dehydrogenase (*zwf*) failed to yield the desired improvement, but instead redirected carbon flux toward cell growth.This indicates that global metabolic network balance must be comprehensively considered in cofactor engineering to avoid adverse effects of flux redistribution on the synthesis of the target product [31]. HpaBC serves as the core enzyme catalyzing HT biosynthesis, and its active site requires Fe^2+^ to maintain catalytic activity. Transcriptomic analysis also revealed that genes associated with Fe–S clusters were significantly upregulated under HT stress, implying that iron ions are involved in cellular stress response and metabolic regulation. Accordingly, a combined strategy using Fe^2+^ and ascorbic acid was adopted, which not only guaranteed the activity of the key enzyme HpaBC, but also effectively alleviated the oxidative degradation of HT and maintained the stability of the fermentation system.This strategy fills the research gap in the coordinated regulation of cofactors and reaction systems in HT biosynthesis, and differs from previous single regulation concepts that only focused on cofactor supply while neglecting system stability [35].

Hydroxytyrosol toxicity constitutes a critical challenge in its microbial production. Most relevant studies to date have been performed in *Saccharomyces cerevisiae*, which exhibits relatively high tolerance to hydroxytyrosol [20,[36], [37], [38]]. Transcriptomic analysis of high-level caffeic acid-producing strains has identified potential transporters that can further improve caffeic acid production. A previous study reported that overexpression of *ycjP* in *E*. *coli* increased caffeic acid titer to 775.7 mg/L, demonstrating that transporter engineering can enhance the biosynthesis of bioactive compounds [39]. A two-phase fermentation strategy to alleviate the growth-inhibitory effect of hydroxytyrosol, using 1-dodecanol as the extractant for in situ removal of hydroxytyrosol from the fermentation broth.The final titer reached 1243 mg/L, representing a 75% improvement compared with the control medium.To date, this remains the only reported case addressing hydroxytyrosol toxicity in practical bioproduction [25]. Results from the present study confirm that hydroxytyrosol inhibits the growth of the host strain, especially when its concentration exceeds 3 g/L, at which cell viability is severely compromised.Transcriptomic analysis identified multiple genes with significantly altered expression levels under hydroxytyrosol stress.Notably, overexpression of *marR* not only increased hydroxytyrosol production but also enhanced strain tolerance, indicating that modulation of stress responses and transport systems contributes to improved product tolerance.RmaH, a MarR family regulator, mediates acid tolerance in *Lactococcus lactis* by regulating genes involved in peptidoglycan modification. MarR family proteins are also involved in various essential physiological processes, including central carbon metabolism, oxidative stress, virulence regulation, and metabolism of diverse aromatic compounds [40]. The *E. coli* MarR family protein MarR identified in this study provides a key target for the future construction of highly tolerant engineered strains [41].

In summary, using *E*. *coli* JNYPQ as the chassis strain, an efficient and genetically stable hydroxytyrosol (HT)-producing strain was constructed by combining metabolic engineering and cofactor engineering strategies.While achieving efficient biosynthesis, this study systematically overcame the critical bottlenecks including precursor metabolic competition, imbalanced cofactor supply and demand, and intracellular product toxicity stress, thereby providing an economically feasible technical scheme for the industrial biomanufacturing of HT. The integrated biosynthesis strategy established in this work-featuring precise precursor partitioning, synergistic cofactor supply, stable regulation of fermentation processes, and enhanced strain tolerance-not only advances technological progress in the biosynthesis of hydroxytyrosol, but also offers a reproducible and scalable systematic engineering paradigm for the efficient microbial production of high value-added phenolic compounds.

Using *E. coli* JNYPQ as the basis, a very stable and efficient hydroxytyrosol production platform was constructed using metabolic engineering and cofactor engineering. These findings provide crucial new knowledge for the consistent production of hydroxytyrosol in *E. coli* cell factories. Future research could concentrate on improving the following elements to significantly boost *E. coli*'s hydroxytyrosol synthesis: (1) Developing HT-tolerant strains with increased hydroxytyrosol resistance through genomic screening or adaptive evolution. (2) Evaluating the robustness of industrial strains while optimizing fermentation to ensure consistent production in large-scale environments. (3) Creating synthetic enzyme scaffolds or fusion proteins to improve substrate channeling effects; directing the development of HpaBC to improve its hydroxytyrosol-specific catalytic activity. (4) Reducing product inhibition and extracting hydroxytyrosol from the fermentation broth in real time by using in situ product separation techniques, such as resin adsorption or organic solvent extraction.

## 5 Conclusions

For this study, a high-yield hydroxytyrosol-producing *E*. *coli* strain was produced by screening the enzymes involved in the hydroxytyrosol biosynthesis route and introducing the corresponding biosynthetic pathway. Branching routes were removed and the precursor supply was raised in order to halt the loss of carbon flux. The copy number of the route enzymes was increased, and significant rate-limiting enzymes were substituted. Because the accumulation of the intermediate byproduct l-DOPA resulted in a considerable loss of metabolic flow, l-amino acid deaminase (LAAD) was introduced to convert l-DOPA into the final product. The overexpression of the *ribHCF* and *pntAB* enhanced the availability of FAD and NADPH, which in turn raised the formation of hydroxytyrosol. Transcriptomic research revealed that increasing the output of hydroxytyrosol and enhancing the tolerance of the transformed strain required the overexpression of *marR*. After 48 h, a 5 L fermenter using glucose as the carbon source produced a hydroxytyrosol titer of 9.25 g/L with a glucose conversion rate of 10.2%, providing fresh data for industrial-scale production.

## CRediT authorship contribution statement

**Jiaojiao Zuo:** Writing – review & editing, Writing – original draft, Validation, Investigation. **Shaolun Zhang:** Writing – review & editing, Methodology. **Wenxiao Huang:** Resources, Investigation. **Jia Liu:** Resources, Investigation. **Cong Gao:** Validation, Formal analysis. **Gui peng Hu:** Methodology. **Wei Song:** Resources, Investigation. **Xiaomin Li:** Resources, Investigation. **Wanqing Wei:** Methodology. **Jing Wu:** Methodology. **Liming Liu:** Validation, Methodology, Formal analysis. **Kaifang Liu:** Writing – review & editing, Supervision, Project administration, Funding acquisition. **Nan Xu:** Writing – review & editing, Supervision, Funding acquisition, Conceptualization.

## Declaration of competing interest

The authors declare that they have no known competing financial interests or personal relationships that could have appeared to influence the work reported in this paper.
