# Supplementary material for: Systems engineering of Escherichia coli for high-level hydroxytyrosol production
Source: Synth Syst Biotechnol. 2026 Jun 1;14:399–412. doi: 10.1016/j.synbio.2026.04.025 (PMC13251646; doi:10.1016/j.synbio.2026.04.025)
Supplement: Multimedia component 1 [file mmc1.docx]

# Systems engineering of *Escherichia coli* for high-level Hydroxytyrosol production

JiaoJiao Zuo^a^, Shaolun Zhang^b,c^, Wenxiao Huang^b,c^, Jia Liu^b,c^, Cong Gao^b,c^, Gui peng Hu^d^, Wei Song^d^, Xiaomin Li^b,c^, Wanqing Wei^b,c^, Jing Wu^d^, Liming Liu^b,c^, Kaifang Liu^b,c*^, Nan Xu^a*^

**^a^** College of Bioscience and BiotechnologyYangzhou University, Yangzhou, China

**^b^** School of Biotechnology, Jiangnan University, Wuxi, 214122, China

**^c^** Key Laboratory of Industrial Biotechnology, Ministry of Education, Jiangnan University, Wuxi 214122, China

**^d^** School of Life Sciences and Health Engineering, Jiangnan University, Wuxi 214122, China

* Corresponding author: State Key Laboratory of Food Science and Technology, Jiangnan University, 1800 Lihu Road, Wuxi 214122, China.

E-mail: mingll@Jiangnan.edu.cn (Liming Liu).

This file includes:

Supplementary Fig. 1 to Supplementary Fig. 4

Supplementary Table 1 to Table 4

# Supplementary Figures

**
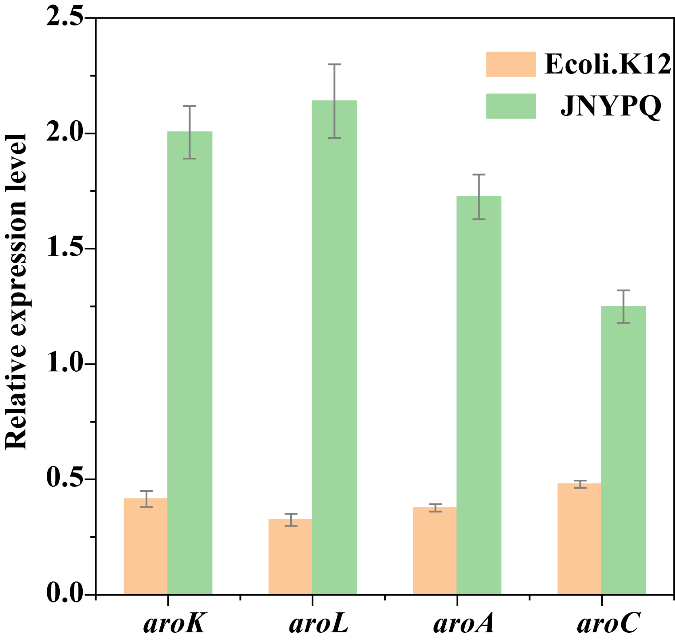
**

## Supplementary Figure 1. RT-qPCR analysis confirmed changes in gene transcription levels

Compared with the starting strain, the expression levels of genes encoding shikimate kinase (*aroK*, *aroL*), 5-enolpyruvylshikimate-3-phosphate synthase (*aroA*), and chorismate synthase (*aroC*) in JNYPQ were upregulated by 4.48-fold, 5.95-fold, 4.28-fold, and 2.88-fold, respectively.


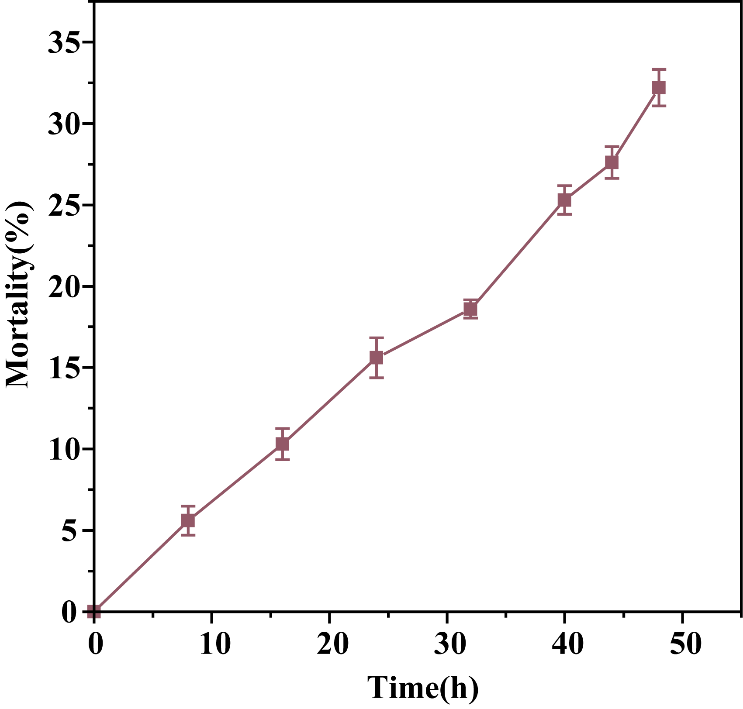


## Supplementary Figure 2. Mortality curve of HT29 strain in shake flask over 48 hours


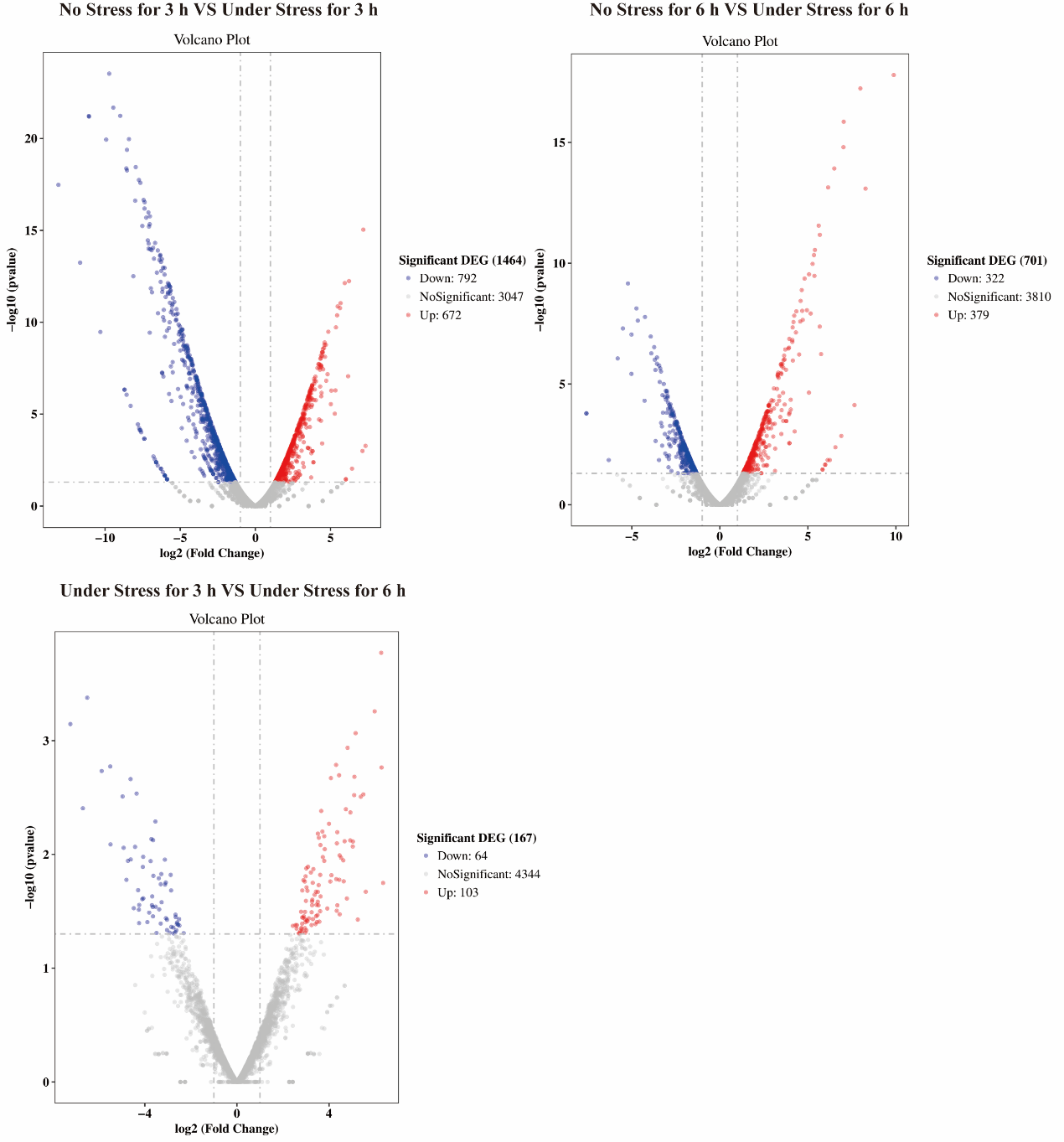


## Supplementary Figure S3. Transcriptome analysis of differentially expressed genes.

Transcriptomics analysis of *E. coli* HT29-2 at 0 g/L and 3 g/L Hydroxytyrosol. Strains HT29-2 were cultured in fermentation media containing 0 g/L and 3 g/L hydroxytyrosol for 3 h and 6 h, followed by transcriptomic sequencing to analyze differentially expressed gene.


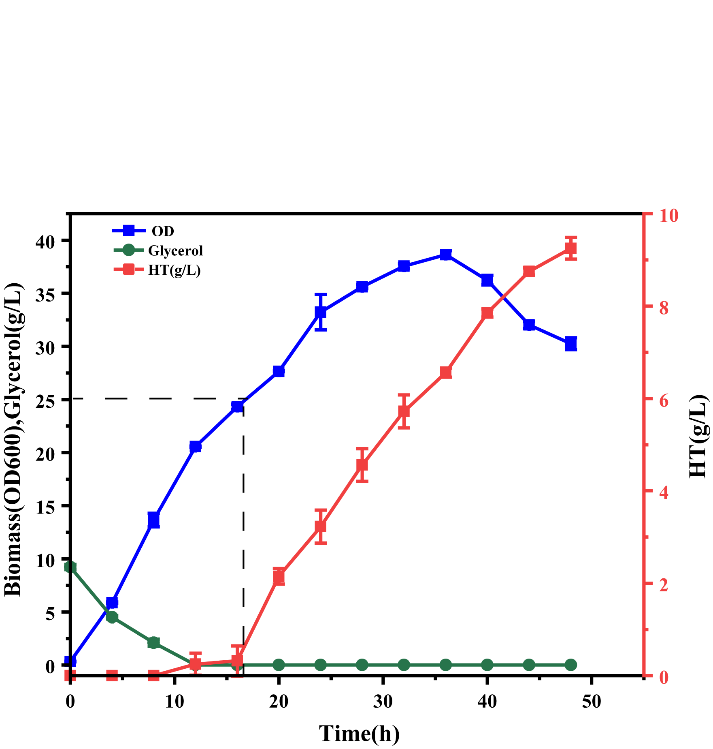


**Supplementary Figure 4**. Glycerol consumption curve of the HT29-2 strain under optimal fermentation conditions, with the dashed line indicating the IPTG addition time .

# Supplementary Tables

## Supplementary Table 1. Strains used in this study.

| Strains | Characteristics | References |  |  |
| --- | --- | --- | --- | --- |
| *E. coli* JM109 | General cloning host | Takara Bio |  |  |
| *E. coli* TOP10 | General cloning host | Takara Bio |  |  |
| *E. coli* BL21(DE3) | For enzyme expression and purification | our lab |  |  |
| *E. coli* JNYPQ | Derivative of *E. coli* K12, capable of producing Phenylalanine, complementation of *tyrA* | our lab |  |  |
| *E. coli* 01 | *E. coli* BL21, pACYC-*ARO10*-*HpaBC*-*ADH6* | This study |  |  |
| *E. coli* 02 | *E. coli* BL21, pACYC- *ARO10*-*HpaBC*-*Yahk* | This study |  |  |
| *E. coli* HT01 | *E. coli* JNYPQ, pACYC- *ARO10*-*HpaBC*-*ADH6* | This study |  |  |
| *E. coli* HT02 | *E. coli* JNYPQ, pET- *ARO10*-*HpaBC*-*ADH6* | This study |  |  |
| *E. coli* HT03 | *E. coli* JNYPQ, pRSF- *ARO10*-*HpaBC*-*ADH6* | This study |  |  |
| *E. coli* HT04 | *E. coli* JNYPQ, pCDF- *ARO10*-*HpaBC*-*ADH6* | This study |  |  |
| *E. coli* HT05 | *E. coli* JNYPQ, pRSF- *ARO10*-*HpaBC*-*ADH6* |  |  |  |
| *E. coli* HT06 | *E. coli* JNYPQ, Δ*pheA*, pRSF- *ARO10*-*HpaBC*-*ADH6* | This study |  |  |
| *E. coli* HT07 | *E. coli* JNYPQ, Δ*pheA*, Δ*cdh*::P_tac_-*aroK*, pRSF-*ARO10*-*HpaBC*-*ADH6* | This study |  |  |
| *E. coli* HT08 | *E. coli* JNYPQ, Δ*pheA*, Δ*cdh::*P_tac_-*aroL*, pRSF-*ARO10*-*HpaBC*-*ADH6* | This study |  |  |
| *E. coli* HT09 | *E. coli* JNYPQ, Δ*pheA*, Δ*cdh::*P_tac_-*aroC*, pRSF-*ARO10*-*HpaBC*-*ADH6* | This study |  |  |
| *E. coli* HT10 | *E. coli* JNYPQ, Δ*pheA*, Δ*cdh::* P_tac_-*tyrA1*, pRSF-*ARO10*-*HpaBC*-*ADH6* | This study |  |  |
| *E. coli* HT11 | *E. coli* JNYPQ, Δ*pheA*, pRSF-*ARO10*-*HpaBC*-*ADH6*, pJ01-*aroK*- *aroC*-*tyrA*1 | This study |  |  |
| *E. coli* HT11-1 | *E. coli* JNYPQ, Δ*pheA*, pRSF-*ARO10*-*HpaBC*-*ADH6*, pJ01-P_J23119-_*aroK*- P_J23119-_*aroC*- P_J23119-_*tyrA*1 | This study |  |  |
|  |  |  |  |  |
| *E. coli* HT11-2 | | | *E. coli* JNYPQ, Δ*pheA*, pRSF-*ARO10*-*HpaBC*-*ADH6*, pJ01-P_J23119-_*aroK*- P_J23119-_*aroC*- P_J23105-_*tyrA*1 | This study |
| *E. coli* HT11-3 | *E. coli* JNYPQ, Δ*pheA*, pRSF-*ARO10*-*HpaBC*-*ADH6*, pJ01-P_J23119-_*aroK*- P_J23119-_*aroC*- P_J23115-_*tyrA*1 | This study |  |  |
| *E. coli* HT11-4 | *E. coli* JNYPQ, Δ*pheA*, pRSF-*ARO10*-*HpaBC*-*ADH6*, pJ01-P_J23119-_*aroK*- P_J23115-_*aroC*- P_J23105-_*tyrA*1 | This study |  |  |
| *E. coli* HT11-5 | *E. coli* JNYPQ, Δ*pheA*, pRSF-*ARO10*-*HpaBC*-*ADH6*, pJ01-P_J23119-_*aroK*- P_J23105-_*aroC*- P_J23119-_*tyrA*1 | This study |  |  |
| *E. coli* HT11-6 | *E. coli* JNYPQ, Δ*pheA*, pRSF-*ARO10*-*HpaBC*-*ADH6*, pJ01-P_J23119-_*aroK*- P_J23105-_*aroC*- P_J23115-_*tyrA*1 | This study |  |  |
| *E. coli* HT11-7 | *E. coli* JNYPQ, Δ*pheA*, pRSF-*ARO10*-*HpaBC*-*ADH6*, pJ01-P_J23119-_*aroK*- P_J23115-_*aroC*- P_J23105-_*tyrA*1 | This study |  |  |
| *E. coli* HT11-8 | *E. coli* JNYPQ, Δ*pheA*, pRSF-*ARO10*-*HpaBC*-*ADH6*, pJ01-P_J23119-_*aroK*- P_J23115-_*aroC*- P_J23115-_*tyrA*1 | This study |  |  |
| *E. coli* HT11-9 | *E. coli* JNYPQ, Δ*pheA*, pRSF-*ARO10*-*HpaBC*-*ADH6*, pJ01-P_J23119-_*aroK*- P_J23105-_*aroC*- P_J23105-_*tyrA*1 | This study |  |  |
| *E. coli* HT11-10 | *E. coli* JNYPQ, Δ*pheA*, pRSF-*ARO10*-*HpaBC*-*ADH6*, pJ01-P_J23105-_*aroK*- P_J23105-_*aroC*- P_J23105-_*tyrA*1 | This study |  |  |
| *E. coli* HT11-11 | *E. coli* JNYPQ, Δ*pheA*, pRSF-*ARO10*-*HpaBC*-*ADH6*, pJ01-P_J23105-_*aroK*- P_J23105-_*aroC*- P_J23115-_*tyrA*1 | This study |  |  |
| *E. coli* HT11-12 | *E. coli* JNYPQ, Δ*pheA*, pRSF-*ARO10*-*HpaBC*-*ADH6*, pJ01-P_J23105-_*aroK*- P_J23105-_*aroC*- P_J23119-_*tyrA*1 | This study |  |  |
| *E. coli* HT11-13 | *E. coli* JNYPQ, Δ*pheA*, pRSF-*ARO10*-*HpaBC*-*ADH6*, pJ01-P_J23105-_*aroK*- P_J23119-_*aroC*- P_J23105-_*tyrA*1 | This study |  |  |
| *E. coli* HT11-14 | *E. coli* JNYPQ, Δ*pheA*, pRSF-*ARO10*-*HpaBC*-*ADH6*, pJ01-P_J23105-_*aroK*- P_J23119-_*aroC*- P_J23105-_*tyrA*1 | This study |  |  |
| *E. coli* HT11-15 | *E. coli* JNYPQ, Δ*pheA*, pRSF-*ARO10*-*HpaBC*-*ADH6*, pJ01-P_J23105-_*aroK*- P_J23115-_*aroC*- P_J23119-_*tyrA*1 | This study |  |  |
|  |  |  |  |  |
| *E. coli* HT11-16 | | *E. coli* JNYPQ, Δ*pheA*, pRSF-*ARO10*-*HpaBC*-*ADH6*, pJ01-P_J23105-_*aroK*- P_J23119-_*aroC*- P_J23115-_*tyrA*1 | This study |  |
| *E. coli* HT11-17 | | *E. coli* JNYPQ, Δ*pheA*, pRSF-*ARO10*-*HpaBC*-*ADH6*, pJ01-P_J23105-_*aroK*- P_J23115-_*aroC*- P_J23115-_*tyrA*1 | This study |  |
| *E. coli* HT11-18 | | *E. coli* JNYPQ, Δ*pheA*, pRSF-*ARO10*-*HpaBC*-*ADH6*, pJ01-P_J23105-_*aroK*- P_J23119-_*aroC*- P_J23119-_*tyrA*1 | This study |  |
| *E. coli* HT11-19 | | *E. coli* JNYPQ, Δ*pheA*, pRSF-*ARO10*-*HpaBC*-*ADH6*, pJ01-P_J23115-_*aroK*- P_J23115-_*aroC*- P_J23115-_*tyrA*1 | This study |  |
| *E. coli* HT11-20 | | *E. coli* JNYPQ, Δ*pheA*, pRSF-*ARO10*-*HpaBC*-*ADH6*, pJ01-P_J23115-_*aroK*- P_J23115-_*aroC*- P_J23105-_*tyrA*1 | This study |  |
| *E. coli* HT11-21 | | *E. coli* JNYPQ, Δ*pheA*, pRSF- ARO10-HpaBC-ADH6, pJ01-P_J23115-_*aroK*- P_J23115-_*aroC*- P_J23119-_*tyrA*1 | This study |  |
| *E. coli* HT11-22 | | *E. coli* JNYPQ, Δ*pheA*, pRSF-*ARO10*-*HpaBC*-*ADH6*, pJ01-P_J23115-_*aroK*- P_J23105-_*aroC*- P_J23115-_*tyrA*1 | This study |  |
| *E. coli* HT11-23 | | *E. coli* JNYPQ, Δ*pheA*, pRSF-*ARO10*-*HpaBC*-*ADH6*, pJ01-P_J23115-_*aroK*- P_J23119-_*aroC*- P_J23115-_*tyrA*1 | This study |  |
| *E. coli* HT11-24 | | *E. coli* JNYPQ, Δ*pheA*, pRSF-*ARO10*-*HpaBC*-*ADH6*, pJ01-P_J23115-_*aroK*- P_J23105-_*aroC*- P_J23119-_*tyrA*1 | This study |  |
| *E. coli* HT11-25 | | *E. coli* JNYPQ, Δ*pheA*, pRSF-*ARO10*-*HpaBC*-*ADH6*, pJ01-P_J23115-_*aroK*- P_J23119-_*aroC*- P_J23115-_*tyrA*1 | This study |  |
| *E. coli* HT11-26 | | *E. coli* JNYPQ, Δ*pheA*, pRSF-*ARO10*-*HpaBC*-*ADH6*, pJ01-P_J23115-_*aroK*- P_J23105-_*aroC*- P_J23105-_*tyrA*1 | This study |  |
| *E. coli* HT11-27 | | *E. coli* JNYPQ, Δ*pheA*, pRSF-*ARO10*-*HpaBC*-*ADH6*, pJ01-P_J23115-_*aroK*- P_J23119-_*aroC*- P_J23119-_*tyrA*1 | This study |  |
| *E. coli* HT12 | | *E. coli* JNYPQ, Δ*pheA*, Δ*ygaY*:: P_J23105_-*aroK*- P_J23105_-*aroC*- P_J23119_-*tyrA*1, pRSF-*ARO10*-*HpaBC*-*ADH6* | This study |  |
| *E. coli* HT13 | | *E. coli* JNYPQ, Δ*pheA*, Δ*pykA,* Δ*ygaY*::P_J23105-_*aroK*- P_J23105-_*aroC*-P_J23119-_*tyrA*1, pRSF-*ARO10*-*HpaBC*-*ADH6* | This study |  |
|  | |  |  |  |
| *E. coli* HT14 | *E. coli* JNYPQ, Δ*pheA*, Δ*pykA,* Δ*feaB,* Δ*ygaY*::P_J23105-_*aroK*- P_J23105-_*aroC*-P_J23119-_*tyrA*1, pRSF-*ARO10*-*HpaBC*-*ADH6* | This study |  |  |
| *E. coli* HT15 | *E. coli* JNYPQ, Δ*pheA*, Δ*pykA,* Δ*feaB,* Δ*mhpB,* Δ*ygaY*::P_J23105-_*aroK*- P_J23105-_*aroC*-P_J23119-_*tyrA*1, pRSF-*ARO10*-*HpaBC*-*ADH6* | This study |  |  |
| *E. coli* HT16 | *E. coli* JNYPQ, Δ*pheA*, Δ*pykA,* Δ*feaB,* Δ*ygaY*::P_J23105-_*aroK*- P_J23105-_*aroC*-P_J23119-_*tyrA*1, pRSF-*ARO10*-*HpaBC*-*ADH6*,Δ*cbrB*::P_J23119-_*ARO10* | This study |  |  |
| *E. coli* HT17 | *E. coli* JNYPQ, Δ*pheA*, Δ*pykA,* Δ*feaB,* Δ*ygaY*::P_J23105-_*aroK*- P_J23105-_*aroC*-P_J23119-_*tyrA*1, pRSF-*ARO10*-*HpaBC*-*ADH6*, Δ*cbrB*::P_J23119-_*ADH6* | This study |  |  |
| *E. coli* HT18 | *E. coli* JNYPQ, Δ*pheA*, Δ*pykA,* Δ*feaB,* Δ*ygaY*::P_J23105-_*aroK*- P_J23105-_*aroC*-P_J23119-_*tyrA*1,pRSF- ARO10-HpaBC-ADH6,Δ*cbrB*::P_J23119-_*HpaBC* | This study |  |  |
| *E. coli* HT19 | *E. coli* JNYPQ, Δ*pheA*, Δ*pykA*, Δ*feaB*, Δ*ygaY*::P_J23105-_*aroK*-P_J23105-_*aroC*-P_J23119-_*tyrA*1,  pRSF-*ARO10*^D331C^-*HpaBC*-*ADH6* | This study |  |  |
| *E. coli* HT20 | | *E. coli* JNYPQ, Δ*pheA*, Δ*pykA*, Δ*feaB*, Δ*ygaY*::P_J23105-_*aroK*-P_J23105-_*aroC*-P_J23119-_*tyrA*1,  pRSF-*ARO10*^D331C^-*HpaBC*-*ADH6-DODC* | This study |  |
| *E. coli* HT21 | *E. coli* JNYPQ, Δ*pheA*, Δ*pykA*, Δ*feaB*, Δ*ygaY*::P_J23105-_*aroK*-P_J23105-_*aroC*-P_J23119-_*tyrA*1,  pRSF-*ARO10*^D331C^-*HpaBC*-*ADH6*-*LAAD* | This study |  |  |
| *E. coli* HT20-1 | *E. coli* JNYPQ, Δ*pheA*, Δ*pykA*, Δ*feaB*, Δ*ygaY*::P_J23105-_*aroK*-P_J23105-_*aroC*-P_J23119-_*tyrA*1,  pRSF-*ARO10*^D331C^-*HpaBC*-*ADH6-DODC* , pETDuet-*TYO* | This study |  |  |
| *E. coli* HT22 | *E. coli* HT20,Δ*rph*:: P_J23119_-*guaA* | This study |  |  |
| *E. coli* HT23 | *E. coli* HT20, Δ*rph*:: P_J23119_-*ribH* | This study |  |  |
| *E. coli* HT24 | *E. coli* HT20, Δ*rph*:: P_J23119_-*ribC* | This study |  |  |
| *E. coli* HT25 | *E. coli* HT20, Δ*rph*:: P_J23119_-*ribF* | This study |  |  |
| *E. coli* HT26 | *E. coli* HT20, Δ*rph*:: P_J23119_-*ribHCF* | This study |  |  |
| *E. coli* HT27 | *E. coli* HT20, Δ*rph*:: P_J23119_-*ribHCF,* Δ*ylbE*:: P_J23119_- *POS5P* | This study |  |  |
| *E. coli* HT28 | *E. coli* HT20, Δ*rph*:: P_J23119_-*ribHCF,* Δ*ylbE*:: P_J23119_- *nadK* | This study |  |  |
| *E. coli* HT29 | *E. coli* HT20, Δ*rph*:: P_J23119_-*ribHCF,* Δ*ylbE*:: P_J23119_- *pntAB* | This study |  |  |
| *E. coli* HT30 | *E. coli* HT20, Δ*rph*:: P_J23119_-*ribHCF,* Δ*ylbE*:: P_J23119_- *GDH* | This study |  |  |
| *E. coli* HT31 | *E. coli* HT20, Δ*rph*:: P_J23119_-*ribHCF,* Δ*ylbE*:: P_J23119_- *zwf* | This study |  |  |
| *E. coli* HT29-1 | *E. coli* HT29, Δ*ydJk*:: P_J23119_- *malG* | This study |  |  |
| *E. coli* HT29-2 | *E. coli* HT29, Δ*ydJk*:: P_J23119_- *marR* | This study |  |  |
| *E. coli* HT29-3 | *E. coli* HT29, Δ*ydJk*:: P_J23119_- *cbl* | This study |  |  |
| *E. coli* HT29-4 | *E. coli* HT29, Δ*ydJk*:: P_J23119_- *iscR* | This study |  |  |
| *E. coli* HT29-5 | *E. coli* HT29, Δ*ydJk*:: P_J23119_- *dctR* | This study |  |  |
| *E. coli* HT29-6 | *E. coli* HT29, Δ*ydJk*:: P_J23119_- *gals* | This study |  |  |
| *E. coli* HT29-7 | *E. coli* HT29, Δ*ydJk*:: P_J23119_- *malF* | This study |  |  |
| *E. coli* HT29-8 | *E. coli* HT29, Δ*ydJk*:: P_J23119_- *lgoR* | This study |  |  |
|  |  |  |  |  |

## Supplementary Table 2. Plasmids used in this study.

| Plasmids | Characteristics | References |
| --- | --- | --- |
| pCas | pMB1 ori, Kan, P_cas_*-*cas9, P_araB_-Red, P_trc_-sgRNA | Lab stock |
| pTargetF | pMB1 ori, Spe^R^, P_J23119_ promoter | Lab stock |
| pACYCDuet | p15A ori, Cmr, P_T7_-laco operator | Lab stock |
| pETuet | pBR322 ori, Amp^R^, P_T7_-laco operator | Lab stock |
| pCDFuet | CDF ori, SmR,P_T7_-laco operator | Lab stock |
| pRSFDuet | RSF ori, Kan,P_T7_-laco operator | Lab stock |
| pJ01 | pMB1 ori, Amp^R^, P_J23119_ promoter | (LI et al., 2022) |
| pET28a | F1 ori, Kan, P_lac_-*lacI*, P_T7_-laco operator, 6×His | Lab stock |
| pTargetF-*feaB* | pMB1 ori, Spe^R^, P_J23119,_ *feaB*-N20 | This study |
| pTargetF-*ygaY* | pMB1 ori, Spe^R^, P_J23119,_ *ygaY*-N20 | This study |
| pTargetF-*rph* | pMB1 ori, Spe^R^, P_J23119,_ *rph*-N20 | This study |
| pTargetF-*ylbE* | pMB1 ori, Spe^R^, P_J23119,_ *ylbE*-N20 | This study |
| pTargetF-*ydJk* | pMB1 ori, Spe^R^, P_J23119,_ *ydJk*-N20 | This study |
| pTargetF-*pykA* | pMB1 ori, Spe^R^, P_J23119,_ *pykA*-N20 | This study |
| pTargetF-*pheA* | pMB1 ori, Spe^R^, P_J23119,_ *pheA*-N20 | This study |
|  | pMB1 ori, Spe^R^, P_J23119,_ *mhpB*-N20 | This study |
| pet28a-senHpaBC | F1 ori, Kan, P_lac_-*lacI*, P_T7_-lac operator-SenHpaBC, 6×His | This study |
| pet28a-KpnHpaBC | F1 ori, Kan, P_lac_-*lacI*, P_T7_-lac operator- KpnHpaBC, 6×His | This study |
| pet28a-EblHpaBC | F1 ori, Kan, P_lac_-*lacI*, P_T7_-lac operator- EblHpaBC, 6×His | This study |
| pet28a-KpnARO10 | F1 ori, Kan, P_lac_-*lacI*, P_T7_-lac operator- KpnARO10, 6×His | This study |
| pet28a-KpaARO10 | F1 ori, Kan, P_lac_-*lacI*, P_T7_-lac operator- KpaRO10, 6×His | This study |
| pet28a-SeaARO10 | F1 ori, Kan, P_lac_-*lacI*, P_T7_-lac operator- SeaARO10, 6×His | This study |

## Supplementary Table 3. Promoters used in this study

| **Promoter** | **Sequence** |
| --- | --- |
| P_J23119_ | TTGACAGCTAGCTCAGTCCTAGGTATAATGCTAGC |
| P_J23105_ | TTTACGGCTAGCTCAGTCCTAGGTACTATGCTAGC |
| P_J23115_ | TTTATAGCTAGCTCAGCCCTTGGTACAATGCTAGC |
| P_tac_ | TTGACAATTAATCATCCGGCTCGTATAATG TTTGCCCTCAACGGTTTTACTCATTGCGATGTGTGT |

## Supplementary Table 4. Primers used in this study

| Description | | Description Sequence(5’-3’) | Application | | |  |  |
| --- | --- | --- | --- | --- | --- | --- | --- |
| *ARO10*-f | | TATAAGAAGGAGATATACATATGGCACCTGTTACAATTGAAAAGTTCGT | | Integration | |  |  |
| *ARO10*-r | | GGTATATCTCCTTCTATTTTTTATTTCTTTTAAGTGCCGCTGCTTCAA | | Integration | |  |  |
| *ADH6*-f | | ACTTAAAAGAAATAAAAAATAGAAGGAGATATACCATGTCTTATCCTGAGAAATTTGAAGGTATCG | | Integration | |  |  |
| *ADH6*-r | | TATTGCTCAGCGGTGGCAGCAGCCTAGGTTAACTAGTCTGAAAATTCTTTGTCGTAGCCGA | | Integration | |  |  |
| *HpaBC*-f | | TTTGTTTAACTTTAATAAGGAGATATACCATGGGCATGAAACCAGAAGATTTCCGCGCCA | | Integration | |  |  |
| *HpaBC*-r | | GAATTTACCATCACGCATGGTATATCTCCTTTTAAATCGCAGCTTCCATTTCCAGCATCA | | Integration | |  |  |
| pACYC-f | | GGCGCGCCGAGCTCGAATTCGGATCCTGGCTGTGGTGATGATGGT | | Integration | |  |  |
| pACYC-r | | ATGCTTAAGTCGAACAGAAAGTAATCGTATTGTACACGGCC | | Integration | |  |  |
| pCDF-f | | AGCTTGTCGACCTGCAGGCGCGCCGAGCTCGAATTCGGATC | | Integration | |  |  |
| pCDF-r | | CTTAAGTCGAACAGAAAGTAATCGTATTGTACACGGCCGC | | Integration | |  |  |
| pRSF-f | | AAGCTGCGATTTAAAAGGAGATATACCATGCGTGATGGTAAATTCGTTGAAT | | Integration | |  |  |
| pRSF-r | | ATGTATATCTCCTTCTTATACT CTTTTCAATTGTAACAGGTGCCAT | | Integration | |  |  |
| pET-f | | GGTATATCTCCTTCTTAAAGTTAAACAAAATTACGTTCTGATAATTCATGCCCAT | | Integration | |  |  |
| pET-r | | AAACCGATCACGATATCCACCCGTAAAAGCTTGCGGCCGCATAATGCTTA | | Integration | |  |  |
| *tyrA*M53I-f | | GCGCGAGGCATCTATTTTGGCCTCGCGTCG | | Integration | |  | |
| *tyrA*M53I-r | | GGCCAAAATAGATGCCTCGCGCTCCG | | Integration | |  |  |
| *tyrA*A354V-f | | GCGATTACGTGCAGCGTTTTCAGAGTGAAAG | | Integration | |  |  |
| *tyrA*A354V-r | | AAACGCTGCACGTAATCGCCGAACCAGTGCTC | | Integration | |  |  |
| *aroK*-f | | GTCCTAGGTATAATGCTAGCTCGCGACAGCTGAAAGAGGAGAAACTGCAGATGGCAGAGAAACGCAATATCTTTC | | Integration | |  |  |
| *aroK*-r | | AGGGAAAAAAAGATGTTAGTTGCTTTCCAGCATGTGAATAATCT | | Integration | |  |  |
| *aroL*-f | | GTCCTAGGTATAATGCTAGCTCGCGACAGCTGAAAGAGGAGAAACTGCAGATGACACAACCTCTTTTTCTGATCG | | Integration | |  |  |
| *aroL*-r | | AGGGAAAAAAAGATGTCAACAATTGATCGTCTGTGCCAGGGCGCT | | Integration | |  |  |
| *aroA*-f | | CTAGGTATAATGCTAGCTCGCGACAGCTGAAAGAGGAGAAACTGCAGATGGAATCCCTGACGTTACAACCCATC | | Integration | |  |  |
| *aroA*-r | | TACAGGGAAAAAAAGATGTCAGGCTGCCTGGCTAATCCGCGCCAGCT | | Integration | |  |  |
| *aroC*-f | | TCCTAGGTATAATGCTAGCTCGCGACAGCTGAAAGAGGAGAAACTGCAGATGGCTGGAAACACAATTGGACAACT | | Integration | |  |  |
| *tyrA1*-r | | CAGGGAAAAAAAGATGTTACTGGCGATTGTCATTCGCCTGACGCA | | Integration | |  |  |
| *malF*-f | | GTCCTAGGTATAATGCTAGCTCGCGACAGCTGAAAGATGCTAGCTCGCGACAGCTGAAAGAGGAGAAACTGCAGATGGATGTCATTAAAAAGAAACATTGGTGGC | | Integration | |  |  |
| *malF*-r | | ACTCTCCTGAAGGAACGTTATCGCTTAATCAAACTTCATTCGCGTGGCT | | Integration | |  |  |
| *dctR*-f | | TGCTAGCTCGCGACAGCTGAAAGAGGAGAAACTGCAGATGTTTCTTATAATTACCAGGGATACGATGT | | Integration | |  |  |
| *dctR*-r | | ACTCTCCTGAAGGAACGTTATCGCTCACACCAGATAATCAATATGCTGATGGCGTA | | Integration | |  |  |
| *gals*-f | | CCTAGGTATAATGCTAGCTCGCGACAGCTGAAAGAGATAATGCTAGCTCGCGACAGCTGAAAGAGGAGAAACTGCAGATGATCACCATTCGTGATGTAGCGCGTC | | Integration | |  |  |
| *gals*-r | | CACTCTCCTGAAGGAACGTTATCGCTTACATCGCCTGATTTGTTGAGTTAGTGAT | | Integration | |  |  |
| *marR*-f | | TCCTAGGTATAATGCTAGCTCGCGACAGCTGAAAGAGGAGAAACTGCAGGTGAAAAGTACCAGCGATCTGTTCAATG | | Integration | |  |  |
| *marR*-r | | AGGTACAGGGAAAAAAAGATGTTACGGCAGGACTTTCTTAAGCAAATACTCA | | Integration | |  |  |
| *bhsA*-f | | TCCTAGGTATAATGCTAGCTCGCGACAGCTGAAAGAGGAGAAACTGCAGATGAAAAACGTAAAAACCCTCATCG | | Integration | |  |  |
| *bhsA*-r | | TCTTTGAGGTACAGGGAAAAAAAGATGTTATTTATAAATTACTGCTGTTCCATGGAGG | | Integration | |  |  |
| *lgoR*-f | | CCTAGGTATAATGCTAGCTCGCGACAGCTGAAAGAGTAGCTCGCGACAGCTGAAAGAGGAGAAACTGCAGATGAGTCGTTCACAAAATTTACGCCACAATG | | Integration | |  |  |
| *lgoR*-r | | ACTCTCCTGAAGGAACGTTATCGCTTAATGAGCATAACGCGTGTTCTCATTAAT | | Integration | |  |  |
| *malG*-f | | CCTAGGTATAATGCTAGCTCGCGACAGCTGAAAGAGGAGAAACTGCAGATGGCAATGGTCCAACCGAAATCGCAAAA | | Integration | |  |  |
| *malG*-r | | AGGGAAAAAAAGATGTTAACCTTTCACACCACCTGCCGTCAGGCCG | | Integration | |  |  |
| *Pos5P*-f | | ATGCTAGCTCGCGACAGCTGAAAGAGGAGAAACTGCAGATGAGTACGTTGGATTCACATTCCCTAAAGTT | | Integration | |  |  |
| *Pos5P*-r | | ATGGATGATCGTCATTGCGCGTTCCGGTTTTTGGTTCAATTTGGAACCTATC | | Integration | |  |  |
| *pntAB*-f | | ATGCTAGCTCGCGACAGCTGAAAGAGGAGAAACTGCAGATGCGAATTGGCATACCAAGAGAACGGTTAAC | | Integration | |  |  |
| *pntAB*-r | | TGATCGTCATTGCGCGTTCCTTACAGAGCTTTCAGGATTGCATCCACGC | | Integration | |  |  |
| *nadK*-f | | TAGCTCGCGACAGCTGAAAGAGGAGAAACTGCAGATGAATAATCATTTCAAGTGTATTGGCATTGT | | Integration | |  |  |
| *nadk*-r | | ATGATCGTCATTGCGCGTTCCTTAGAATAATTTTTTTGACCAGCCGAGC | | Integration | |  |  |
| GDH-f | | TGCTAGCTCGCGACAGCTGAAAGAGGAGAAACTGCAGATGTATCCGGATTTAAAAGGAAAAGTCGTCGCTA | | Integration | |  |  |
| GDH-r | | GGATGGATGATCGTCATTGCGCGTTCCTTAACCGCGGCCTGCCTGGAATGAA | | Integration | |  |  |
| *rph*-N20-f | | CATCATTGAAGTGCAGGGGAGTTTTAGAGCTAGAAATAGCAAGTTAAAA | | Integration | |  |  |
| *rph*-N20-r | | CCCCTGCACTTCAATGATGACTAGTATTATACCTAGGACTGAGCTAGCT | | Integration | |  |  |
| *ygaY*-N20-f | | TGTCTCGATGACCTTACTGGGTTTTAGAGCTAGAAATAGCAAGTTAAAA | | Integration | |  |  |
| *ygaY*-N20-r | | CCCAGTAAGGTCATCGAGACAACTAGTATTATACCTAGGACTGAGCTAGC | | Integration | |  |  |
| *ylbE*-N20-f | | ACGCCGGGCCGCCAATGCGCGTTTTAGAGCTAGAAATAGCAAGTTAAAA | | Integration | |  |  |
| *ylbE*-N20-r | | GCGCATTGGCGGCCCGGCGTACTAGTATTATACCTAGGACTGAGCTA | | Integration |  |  |  |
| *ydJk*-N20-f | | GCAATAACCACAGGCGGCAAGTTTTAGAGCTAGAAATAGCAAGTTAAA | | Integration | | |  |
| *ydJk*-N20-r | TTGCCGCCTGTGGTTATTGCACTAGTATTATACCTAGGACTGAGCTAG | | | Integration | | |  |
| *cbrB*-N20-f | ATTATCACCCAAAGAACCAGGTTTTAGAGCTAGAAATAGCAAGTTAAAAT | | | Integration | | |  |
| *cbrB*-N20-r | GGTTCTTTGGGTGATAATACTAGTATTATACCTAGGACTGAGCTAGC | | | Integration | | |  |
| *pheA*-N20-f | AGATTCCGTATTAACTCAGCGTTTTAGAGCTAGAAATAGCAAGTTAAAA | | | Integration | | |  |
| *pheA*-N20-r | CGCTGAGTTAATACGGAATCTACTAGTATTATACCTAGGACTGAGCTA | | | Integration | | |  |
| *pykA*-N20-f | GTGTTCACCGAAGTCACCGTGTTTTAGAGCTAGAAATAGCAAGTTAAAAT | | | Integration | | |  |
| *pykA*-N20-r | CACGGTGACTTCGGTGAACACACTAGTATTATACCTAGGACTGAGCTAGC | | | Integration | | |  |
| *feaB*-N20-f | TTTGTCTCGCGTCGCTGGGCGTTTTAGAGCTAGAAATAGCAAGTTAAAA | | | Integration | | |  |
| *feaB*-N20-r | GCCCAGCGACGCGAGACAAAACTAGTATTATACCTAGGACTGAGCTAGC | | | Integration | | |  |
| *mhpB*-N20-f | GGCATTCTCCCCTGAACTGGGTTTTAGAGCTAGAAATAGCAAGTTAAAATA | | | Integration | | |  |
| *mhpB*-N20-r | CTAAAACCCAGTTCAGGGGAGAATGCCACTAGTATTATACCTAGGACTGAGC | | | Integration | | |  |
